# The Cancer Genome Atlas Comprehensive Molecular Characterization of Renal Cell Carcinoma

**DOI:** 10.1016/j.celrep.2018.03.075

**Published:** 2018-04-03

**Authors:** Christopher J. Ricketts, Aguirre A. De Cubas, Huihui Fan, Christof C. Smith, Martin Lang, Ed Reznik, Reanne Bowlby, Ewan A. Gibb, Rehan Akbani, Rameen Beroukhim, Donald P. Bottaro, Toni K. Choueiri, Richard A. Gibbs, Andrew K. Godwin, Scott Haake, A. Ari Hakimi, Elizabeth P. Henske, James J. Hsieh, Thai H. Ho, Rupa S. Kanchi, Bhavani Krishnan, David J. Kwaitkowski, Wembin Lui, Maria J. Merino, Gordon B. Mills, Jerome Myers, Michael L. Nickerson, Victor E. Reuter, Laura S. Schmidt, C. Simon Shelley, Hui Shen, Brian Shuch, Sabina Signoretti, Ramaprasad Srinivasan, Pheroze Tamboli, George Thomas, Benjamin G. Vincent, Cathy D. Vocke, David A. Wheeler, Lixing Yang, William T. Kim, A. Gordon Robertson, The Cancer Genome Atlas Research Network, Paul T. Spellman, W. Kimryn Rathmell, W. Marston Linehan

**Affiliations:** 1 Urologic Oncology Branch, National Cancer Institute, Center for Cancer Research, Bethesda, MD 20892, USA; 2 Vanderbilt University School of Medicine, Nashville, TN 37232, USA; 3 Van Andel Research Institute, Grand Rapids, MI 49503, USA; 4 Lineberger Comprehensive Cancer Center, University of North Carolina at Chapel Hill, Chapel Hill, NC 27599, USA; 5 Memorial Sloan Kettering Cancer Center, New York, NY 10065, USA; 6 Canada’s Michael Smith Genome Sciences Centre, Vancouver, BC V5Z 4S6, Canada; 7 The University of Texas MD Anderson Cancer Center, Houston, TX 77030, USA; 8 The Broad Institute of Massachusetts Institute of Technology and Harvard University, Cambridge, MA 02142, USA; 9 Dana-Farber Cancer Institute, Boston, MA 02215, USA; 10 Baylor College of Medicine, Houston, TX 77030, USA; 11 University of Kansas Medical Center, Kansas City, KS 66206, USA; 12 Brigham and Women’s Hospital, Boston, MA 02115, USA; 13 Washington University School of Medicine, St. Louis, MO 63110, USA; 14 Mayo Clinic Arizona, Phoenix, AZ 85054, USA; 15 Laboratory of Pathology, Center for Cancer Research, National Cancer Institute, Bethesda, MD 20892, USA; 16 Centura Health, Centennial, CO 80112, USA; 17 Division of Cancer Epidemiology and Genetics, National Cancer Institute, Bethesda, MD 20892, USA; 18 Basic Science Program, Leidos Biomedical Research, Inc. Frederick National Laboratory of Cancer Research, Frederick, MD 21702, USA; 19 Leukemia Therapeutics LLC., Hull, MA 02045, USA; 20 Yale University, New Haven, CT 06520, USA; 21 Oregon Health & Science University, Portland, OR 97239, USA; 22 Harvard Medical School, Boston, MA 02115, USA; 23 Lead Contact

## Abstract

Renal cell carcinoma (RCC) is not a single disease, but several histologically defined cancers with different genetic drivers, clinical courses, and therapeutic responses. The current study evaluated 843 RCC from the three major histologic subtypes, including 488 clear cell RCC, 274 papillary RCC, and 81 chromophobe RCC. Comprehensive genomic and phenotypic analysis of the RCC subtypes reveals distinctive features of each subtype that provide the foundation for the development of subtype-specific therapeutic and management strategies for patients affected with these cancers. Somatic alteration of *BAP1*, *PBRM1*, and *PTEN* and altered metabolic pathways correlated with subtype-specific decreased survival, while *CDKN2A* alteration, increased DNA hypermethylation, and increases in the immune-related Th2 gene expression signature correlated with decreased survival within all major histologic subtypes. CIMP-RCC demonstrated an increased immune signature, and a uniform and distinct metabolic expression pattern identified a subset of metabolically divergent (MD) ChRCC that associated with extremely poor survival.

## Graphical abstract

**In Brief** Ricketts et al. find distinctive features of each RCC subtype, providing the foundation for development of subtypespecific therapeutic and management strategies. Somatic alteration of *BAP1*, *PBRM1*, and metabolic pathways correlates with subtype-specific decreased survival, while *CDKN2A* alteration, DNA hypermethylation, and Th2 immune signature correlate with decreased survival within all subtypes.

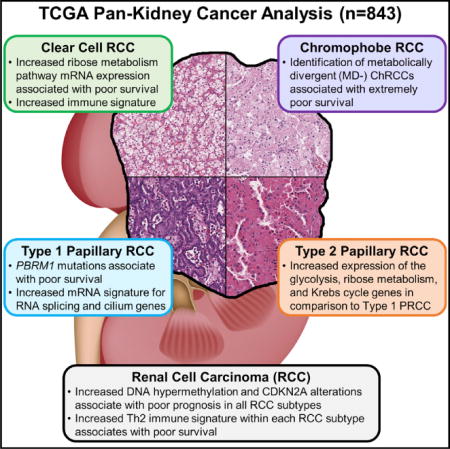

## INTRODUCTION

Renal cell carcinoma (RCC) affects nearly 300,000 individuals worldwide annually and is responsible for more than 100,000 deaths each year. Our understanding of RCC has evolved over the past 40 years, from considering RCC as a single entity to our current understanding that RCC is made up of many different subtypes of renal cancer, each with different histology, distinctive genetic and molecular alterations, different clinical courses, and different responses to therapy (Linehan, 2012; Linehan et al., 2010; Moch et al., 2016). The canonical classification of RCC consists of three major histologic RCC subtypes (Hsieh et al., 2017; Linehan et al., 2006; Moch et al., 2016). Clear cell renal cell carcinoma (ccRCC) is the most common subtype (~75%); papillary renal cell carcinoma (PRCC) accounts for 15%–20% and is subdivided into types 1 and 2; and chromophobe renal cell carcinoma (ChRCC) represents ~5% of RCC.

The Cancer Genome Atlas (TCGA) Research Network has conducted a series of comprehensive molecular characterizations in distinctive histologic types of cancers including ccRCC, ChRCC, and PRCC (Cancer Genome Atlas Research Network, 2013; Cancer Genome Atlas Research Network et al., 2016; Davis et al., 2014). These studies revealed a remodeling of cellular metabolism in ccRCC involving downregulation of Krebs cycle genes, upregulation of pentose phosphate pathway genes, and decreased AMPK in higher-stage, high-grade, and low-survival disease. A distinct PRCC subtype was identified that was characterized by a CpG island methylator phenotype (CIMP-RCC) and associated with early-onset disease, poor survival, and germline or somatic mutation of the fumarate hydratase (*FH*) gene, and a subset of ChRCC with genomic rearrangements within the *TERT* promoter region was identified that correlated with highly elevated *TERT* expression and manifestation of kataegis, uncovering a distinct mechanism of *TERT* upregulation in cancer. A previous study by Chen et al. (2016) compared all available kidney tumor samples available within TCGA irrelevant of histologic type using cluster analysis of the multi-platform genetic and genomic data to show that the majority of the histologic subtypes could be reconstituted. In addition, this study identified samples that fell outside of the major subtypes and identified several mutation, methylation, and immune expression profiles that correlated with histologic subtypes within the complete TCGA kidney cohort.

The importance of histology cannot be understated in the study of RCC. To highlight the most meaningful somatic alterations in the entire cohort and within each major histologic subtype, we performed an integrated comparative genomic analysis of all available histologically confirmed TCGA samples of ccRCC, PRCC, and ChRCC to identify shared and subtype-specific molecular features that will provide the foundation for the development of disease-specific therapeutic approaches and prognostic biomarkers for RCC.

## RESULTS

### Evaluation of RCC Histologic Subtypes

In total, 894 samples of kidney cancer were initially submitted to TCGA and were available for analysis, including 537 ccRCC, 291 PRCC, and 66 ChRCC. The initial TCGA analyses of each RCC subtype had excluded several samples due to inconsistent/incorrect histologic classification or therapy prior to sample collection. This included the removal of a small number of samples, such as transitional cell carcinomas, that are kidney cancers that are not classified as RCCs. Additional samples not utilized in previous studies were also re-evaluated by histologic review and removed if considered inappropriate and 15 samples originally submitted as ccRCC were reclassified as ChRCC. This resulted in a final cohort of 843 TCGA-RCC consisting of 488 ccRCC, 274 PRCC, and 81 ChRCC. The 274 PRCC were further divided into four subgroups consisting of 160 type 1 PRCC, 70 type 2 PRCC, 34 unclassified PRCC, and 10 CpG island methylator phenotype-associated (CIMP)-RCC (Table S1).

### Comparison of Major RCC Histologic Subtypes

**Figure 1:**
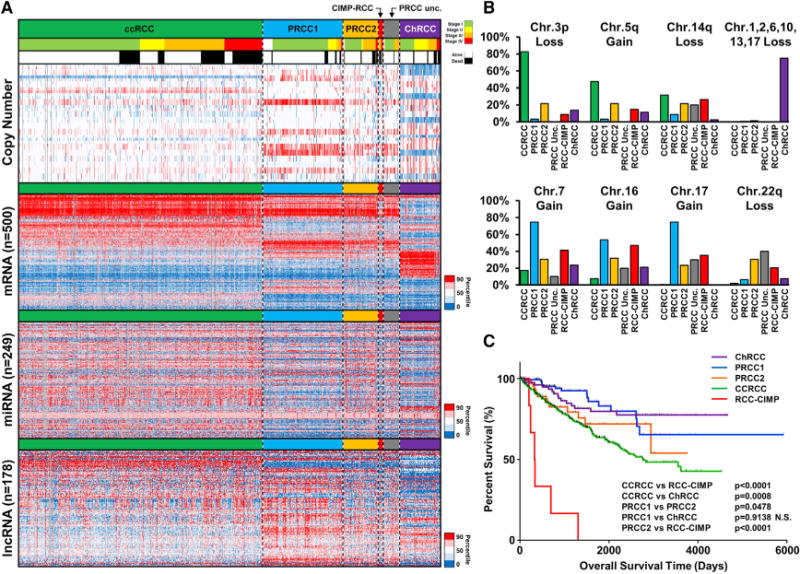
Comparison of RCC Histologic Subtypes (A) Heatmap representation of chromosomal copy number and RNA expression profiles between the different histologic RCC subtypes. Chromosomal copy number data are ordered by chromosomal arm in descending order (red, gain; blue, loss). The relative RNA expression was assessed for the most variable probes within the complete RCC cohort for either mRNA (n = 500), miRNA (n = 249), or lncRNA (n = 178) (red, increased; blue, decreased). RCC samples were arrayed left to right based on histologic subtype (ccRCC, green; type 1 PRCC, light blue; type 2 PRCC, orange; unclassified [Unc.] PRCC, gray; CIMP-RCC, red; ChRCC, purple), then tumor stage (stage I, light green; stage II, yellow; stage III, orange; stage IV, red), and then vital status (alive, white; deceased, black). (B) Percentage of chromosomal copy number alterations between the different histologic RCC subtypes. (C) Differences in patient overall survival between the different histologic RCC subtypes (log-rank p value).

Initial comparison of these RCC was performed using chromosomal copy number profiles, mRNA, miRNA, and lncRNA expression profiles and visualized in a heatmap with the RCCs ordered by histologic subtype, then stage, then vital status (Figure 1A). Clear cell RCC demonstrated significant loss of chromosome 3p and gain of 5q, type 1 PRCC demonstrated gains of chromosomes 7 and 17, and ChRCC demonstrated a pattern of chromosomal losses that included 1, 2, 6, 10, 13, and 17 (Figure 1B). These data confirmed previous observations concerning the copy number patterns within the different RCC histologic subtypes, and somatically gained alterations in chromosomal copy number patterns provide the clearest distinction between subtypes. While specific patterns of copy number alteration were not observed in the CIMP-RCC or the type 2 PRCC, both demonstrated an increased loss of chromosome 22 that encodes *NF2* from the HIPPO pathway and *SMARCB1*, a fundamental component of the SWI/SNF complex, and the CIMP-RCC had loss of chromosome 13q at a similar rate to ChRCC (60% versus 61.3%) that encodes *RB1* and *BRCA2* (Figure 1B). Analysis of RNA expression across the combined cohort demonstrated distinct mRNA, miRNA, and lncRNA clusters that associated with each histologic RCC type. Two mRNA, three miRNA, and five lncRNA clusters were enriched in ccRCC, while two mRNA, two miRNA, and two lncRNA clusters represented the majority of the PRCC (Figures S1A–S1C). The ChRCC samples demonstrated a distinct uniformity by being present in a single cluster for each RNA type, while the CIMP-RCC had a distinct mRNA cluster and shared a lncRNA cluster with the ChRCC.

### Survival Differences across the Major RCC Histologic Subtypes

The variation between the RCC histologic subtypes extended to survival outcomes (Figure 1C). Previously, CIMP-RCC was found to have the poorest PRCC survival but now demonstrated the worst survival of all RCC subtypes, including ccRCC (p < 0.0001). Clear cell RCC demonstrated the next poorest survival when compared to all other RCC subtypes, while type 1 PRCC and ChRCC associated with the best survival that was statistically indistinguishable (p = 0.9138). These histologic-specific differences in survival and the uneven representation of each histologic subtype within the cohort produces a potential confounding factor for survival associations evaluated across the entire cohort. With clear distinctions between the histologic subtypes established, survival associations within histologic subtypes are likely to be more relevant than those across the entire cohort.

### Gene and Pathway Alteration Associates with Survival in Specific RCC Subtypes

**Figure 2:**
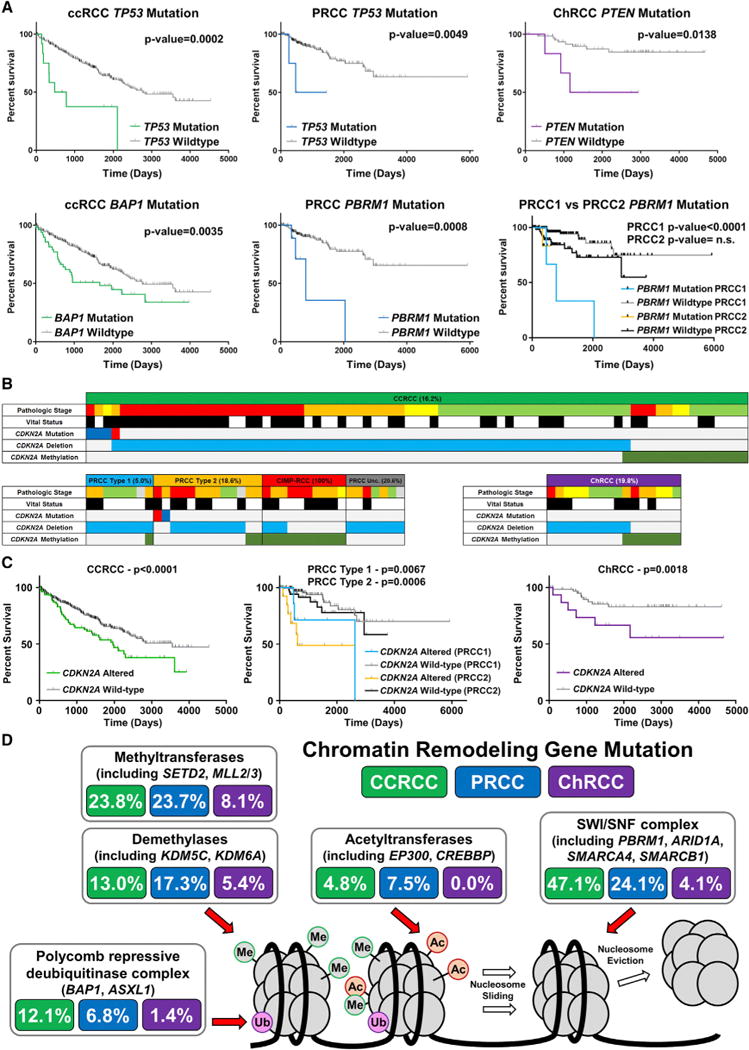
Gene and Pathway Alteration Associates with Survival Predictions in Specific RCC Subtypes (A) Differences in patient overall survival within histologic RCC subtypes (ccRCC, green; PRCC, blue; ChRCC, purple) dependent upon gene mutation (log-rank p value). (B) Oncoprints for *CDKN2A* gene deletions, hypermethylation, and mutations for the histologic RCC subtypes (ccRCC, green; type 1 PRCC, light blue; type 2 PRCC, orange; Unc. PRCC, gray; CIMP-RCC, red; ChRCC, purple). Mutations were segregated into nonsense (red) and missense (blue). (C) Differences in patient overall survival within the histologic RCC subtypes (ccRCC, green; type 1 PRCC, light blue; type 2 PRCC, orange; ChRCC, purple) dependent upon *CDKN2A* alteration (log-rank p value). (D) Chromatin remodeling pathway mutation frequency within histologic RCC subtypes (ccRCC, green; PRCC, blue; ChRCC, purple). Abbreviations: Me, histone methylation; Ac, histone acetylation; Ub, histone ubiquitination.

Previous analyses of each histologic RCC subtype had identified a combined total of 16 significantly mutated genes (SMGs) including 9 associated with ccRCC, 11 associated with PRCC, and 2 associated with ChRCC (Figure S2A; Cancer Genome Atlas Research Network, 2013; Cancer Genome Atlas Research Network et al., 2016; Davis et al., 2014). Analysis across RCC types revealed that *TP53* and *PTEN* were the only SMGs shared by ccRCC, PRCC, and ChRCC (2.6% and 4.5%, 1.5% and 3.4%, and 31.1% and 8.1%, respectively). Across the entire cohort, neither *TP53* nor *PTEN* correlated with poor survival, but histologic-specific analysis demonstrated that *TP53* mutation correlated with decreased survival in ccRCC (p = 0.0002) and PRCC (p = 0.0049), while *PTEN* mutation correlated with decreased survival in ChRCC (p = 0.0138) (Figures 2A and S2B). Clear cell RCC and PRCC, but not ChRCC, shared three chromatin remodeling SMGs: *PBRM1* (38.0% and 4.5%, respectively), *SETD2* (13.2% and 6.4%, respectively), and *BAP1* (11.0% and 5.6%, respectively). While *BAP1* mutation correlated with decreased survival across the entire cohort (p = 0.0002) and within the ccRCC group (p = 0.0035), *BAP1* mutation did not correlate with survival in PRCC or ChRCC. Similarly, *PBRM1* mutation, which has been shown to not correlate with survival in ccRCC, was found to correlate with decreased survival in PRCC (p = 0.0008) that was specific to type 1 PRCC (p < 0.0001) (Figures 2A and S2B). *CDKN2A* mutation, hypermethylation, or deletion was found in 15.8% of tumors, with alterations in each RCC subtype accounting for 16.2% of ccRCC, 5.0% of type 1 PRCC, 18.6% of type 2 PRCC, 100% of CIMP-PRCC, and 19.8% of ChRCC (Figure 2B). Loss of the region of chromosome 9p encoding *CDKN2A* was the most frequent event across the cohort (11.7%), followed by promoter hypermethylation (4.2%) and mutation (0.7%) (Table S1). *CDKN2A* alteration provided the sole example of a change that correlated with decreased survival across the entire cohort (p < 0.0001) and in each major histologic subtype, ccRCC (p < 0.0001), type 1 PRCC (p = 0.0067), type 2 PRCC (p = 0.0006), and ChRCC (p = 0.0018) (Figure 2C).

Eight SMGs were frequently mutated (≥ 2.0%) in more than one RCC subtype. Mutation of at least 1 of the 16 SMGs was found in 81% of ccRCC, 39.1% of PRCC, and 43.2% of ChRCC (Figure S2A). While the overall mutation rate for ChRCC was found to be significantly less than either ccRCC or PRCC (p = 0.0254 and p < 0.0001, respectively), the PRCC mutation rate was higher than ccRCC (p < 0.0001) (Figure S2C). Within PRCC, the most aggressive subtype, CIMP-RCC, was found to have the lowest overall rate of mutation. Pathogenic SMG mutations were not detected in several tumors, particularly PRCC and ChRCC. Several SMGs were members of pathways that contained genes mutated at lower frequencies. In the VHL/HIF pathway, *TCEB1* and *CUL2* mutations in ccRCC were mutually exclusive with *VHL* mutation (Figure S2D). HIPPO and NRF2/ARE pathway mutations were present in both PRCC (9.0% and 7.9%, respectively) and ccRCC (3.9% and 3.2%, respectively) (Figure S2D). While chromatin remodeling pathway gene mutations were notably frequent in both ccRCC (69.3%) and PRCC (53.0%), they were less common in ChRCC (14.9%) (Figure S2D and Table S2). Mutations of SWI/SNF complex genes, including *PBRM1*, *ARID1A*, and *SMARCA4*, were the most common chromatin remodeling complex alterations within ccRCC (47.1%), followed by mutation of the histone methyltransferases including *SETD2* and *MLL3* (23.8%), the histone demethylases including *KDM5C* (13.0%), the BAP1/ASXL1 histone de-ubiquitinase complex (12.1%), and the histone acetyltransferases (4.8%), compared with frequencies of 24.1%, 23.7%, 17.3%, 6.8%, and 7.5%, respectively, in PRCCs (Figure 2D). Chromatin remodeling gene mutations were more frequent in type 2 PRCC (55.3%) than in type 1 PRCC (40.6%). While mutations of the PI3K/AKT pathway were frequent both across (14.6%) as well as within each RCC subtype (16.2% of ccRCC, 9.8% of PRCC, and 18.9% of ChRCC), they correlated with decreased survival only in ChRCC (p = 0.0018) (Figures S2D and S2E and Table S2).

Mitochondrial (mt) DNA mutation analysis, which was previously performed only in ChRCC (Davis et al., 2014), was conducted in a representative number of tumors from all RCC subtypes. Nonsense or missense mutations in mitochondria-encoded genes with high heteroplasmy (>75%) as well as frameshift mutations with >50% heteroplasmy were considered significant. Mitochondrial DNA mutations were found in 13% of 62 ccRCC, 33% of 99 PRCC (with similar frequencies for type 1 and type 2), and 20% of 65 ChRCC. High-heteroplasmy truncating (nonsense or frameshift) mutations were enriched in ChRCC (14%) compared to PRCC (6%) or ccRCC (2%) (Figure S2F) and mtDNA copy number was increased in ccRCC, PRCC, and ChRCC that carried mtDNA mutations (p = 0.0036, p = 0.0036, and p = 0.0029, respectively) (Figure S2F).

### Hypermethylation Correlates with Decreased Survival

**Figure 3:**
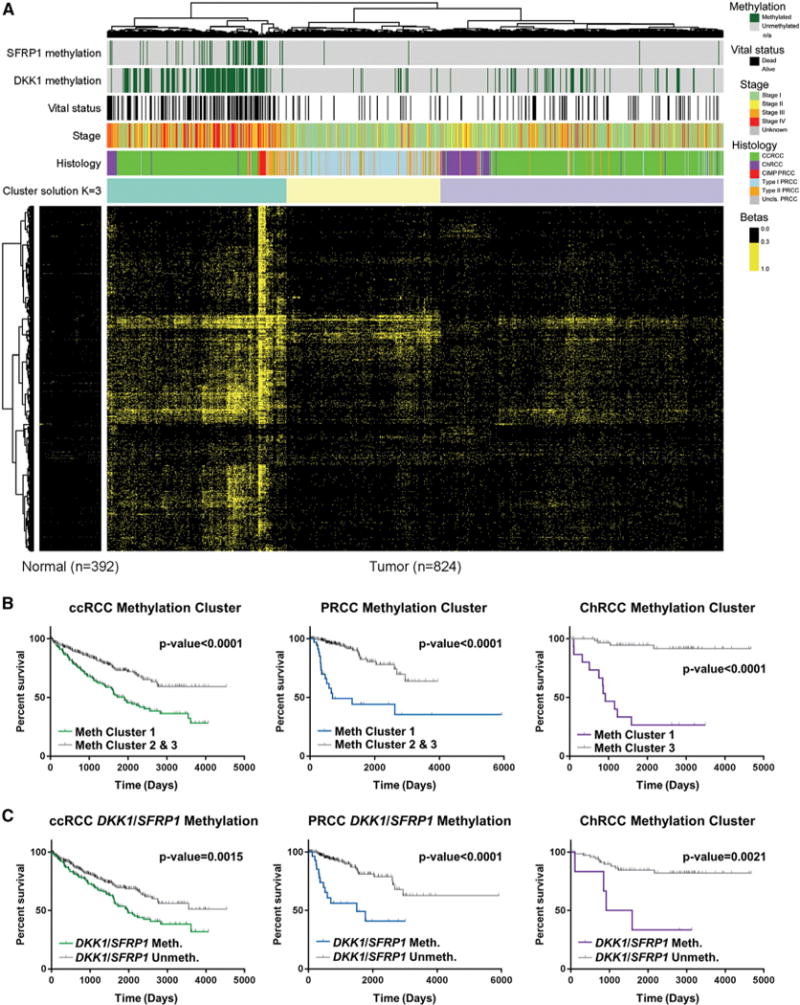
Hypermethylation Patterns Associate with Survival Predictions (A) Heatmap representation of the clustering of 1,532 highly variable DNA methylation probes that were unmethylated in the normal tissues. A methylation b-value R 0.3 was considered hypermethylated. Tumors were annotated for histologic RCC subtype (ccRCC, green; type 1 PRCC, light blue; type 2 PRCC, orange; Unc. PRCC, gray; CIMP-RCC, red; ChRCC, purple), tumor stage (stage I, light green; stage II, yellow; stage III, orange; stage IV, red), vital status (alive, white; deceased, black), and *DKK1* (cg07684796) and *SFRP1* (cg15839448) hypermethylation (hypermethylated, dark green). (B) Differences in patient overall survival within the histologic RCC subtypes (ccRCC, green; PRCC, blue; ChRCC, purple) dependent upon methylation cluster (log-rank p value). (C) Differences in patient overall survival within ccRCC and ChRCC tumors (ccRCC, green; ChRCC, purple) dependent upon hypermethylation of either *SFRP1* or *DKK1* (log-rank p value).

Previous analyses of methylation by Chen et al. (2016) had demonstrated that a subset of the DNA methylation probes within the RCC samples highlighted the differences in cell of origin for the major RCC histologic subtypes. This subset of probes was subsequently used to evaluate hypermethylation patterns within the samples but was potentially confounded by the difference in tumor origin. While hypermethylated ccRCC and PRCC samples were identified, no hypermethylated ChRCC samples were observed. An analysis limited to probes that are unmethylated in all normal tissues identified in 1,532 variably hypermethylated markers that identified a cluster of 240 RCCs with increased DNA hypermethylation (methylation cluster 1) that associated with significantly poorer survival (p < 0.0001) (Figure 3A and Table S1). This cluster consisted of the 10 CIMP-RCC, 182 ccRCC (37.3%), 23 type 2 PRCC (32.9%), 16 ChRCC (19.8%), and a small number of type 1 and unclassified PRCC. The remaining two clusters, one containing type 1 and type 2 PRCC (methylation cluster 2) and the other containing ccRCC and ChRCC (methylation cluster 3), had similar survival. In contrast to the distinct CIMP-RCC tumors that had notably high levels of DNA hypermethylation, the remainder of methylation cluster 1 had a less pronounced increase in hypermethylation across the genome. Histologic subtype-specific analysis confirmed decreased survival with the increased hypermethylation pattern in every major RCC histologic subtype (all p < 0.0001) (Figure 3B). Within the PRCC tumors, this correlation remained significant after excluding the CIMP-RCC from the PRCC tumors (p < 0.0001) and when type 1 PRCC (p = 0.0328) and type 2 PRCC (p = 0.0314) were independently evaluated (Figures 3B and S3A). Increased hypermethylation was associated with higher-stage disease in ccRCC, PRCC (with or without CIMP), and ChRCC (all p < 0.0001) and was associated with *SETD2* mutation in ccRCC (p < 0.0001), either *PBRM1* mutation or *SETD2* mutation in type 2 PRCC (p = 0.0053, p = 0.0270, respectively), and *TP53* mutation in ChRCC (p = 0.0119) (Figure S3B). Genes represented by the 1,532 probes that characterized the hypermethylated cluster were enriched for genes in the WNT pathway. Previous studies have identified hypermethylation of the WNT pathway regulatory genes, *SFRP1* and *DKK1*, in ccRCC (Ricketts et al., 2014). Increased methylation of probes for these two genes (*DKK1*, cg07684796; *SFRP1*, cg15839448) was observed in the methylated cluster 1 samples (Figure S3C), and hypermethylation of either of these genes correlated with poorer survival in ccRCC, PRCC, and ChRCC (p = 0.0015, p < 0.0001, and p = 0.0021, respectively) and in PRCC excluding the CIMP-RCC tumors (p = 0.0035) (Figures 3C and S3D).

### Specific mRNA Signatures Associate with RCC Histologic Subtypes

**Figure 4:**
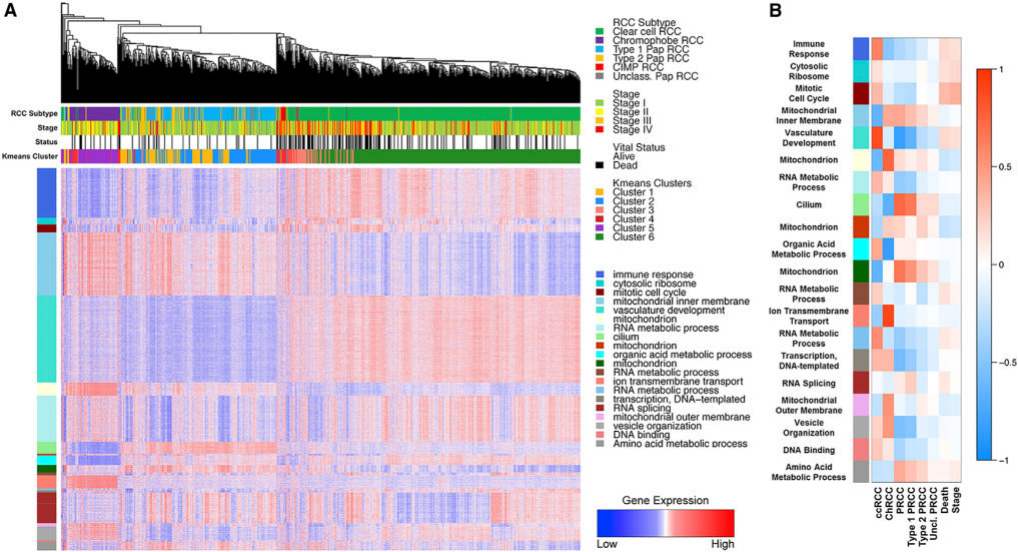
RCC Histologic Subtypes Associate with Specific mRNA Signatures (A) Heatmap representation of the comparison of mRNA expression signatures for major cellular processes between the different histologic RCC subtypes (ccRCC, green; type 1 PRCC, light blue; type 2 PRCC, orange; Unc. PRCC, gray; CIMP-RCC, red; ChRCC, purple). Tumor stage (stage I, light green; stage II, yellow; stage III, orange; stage IV, red) and vital status (alive, white; deceased, black) are indicated above the heatmap. (B) Heatmap representation showing the relationship between gene expression modules and clinical features. Red heatmap shading indicates a positive correlation between a gene module and a clinical feature and blue heatmap shading represents a negative correlation.

A weighted gene co-expression network analysis (WGCNA), performed to identify sets (modules) of highly correlated genes and to assess their relationships to clinical variables and biological functions, revealed several gene modules that differentiated the RCCs by histology, stage, or survival status (Figure 4). Clear cell RCC showed the expected increase in expression of the vasculature development signature, due to activation of the VHL/HIF pathway, and the previously observed increase in immune response signature (p = 4 × 10^−86^) in comparison to PRCC and ChRCC (Figure 4B). The RNA metabolic process and the mitotic cell cycle signature was specifically increased in ccRCC (p = 5 × 10^−26^ and p = 5 × 10^−25^, respectively), while an increased amino acid metabolic process signature (p = 4 × 10^−35^) and retention of cilium signature (p = 3 × 10^−140^) was unique to PRCC (Figure 4B). In ChRCC, an increased ion transmembrane transport signature was observed (Figure 4). Subtype analysis of PRCC revealed an increased RNA splicing signature in type 1 PRCC (p = 2 × 10^−12^) compared to type 2 PRCC, while the cilium signature was significantly higher in the type 1 PRCC (p = 8 × 10^−101^) than in the type 2 PRCC (p = 4 × 10^−7^).

### Metabolic Gene Expression Associates with Survival

**Figure 5:**
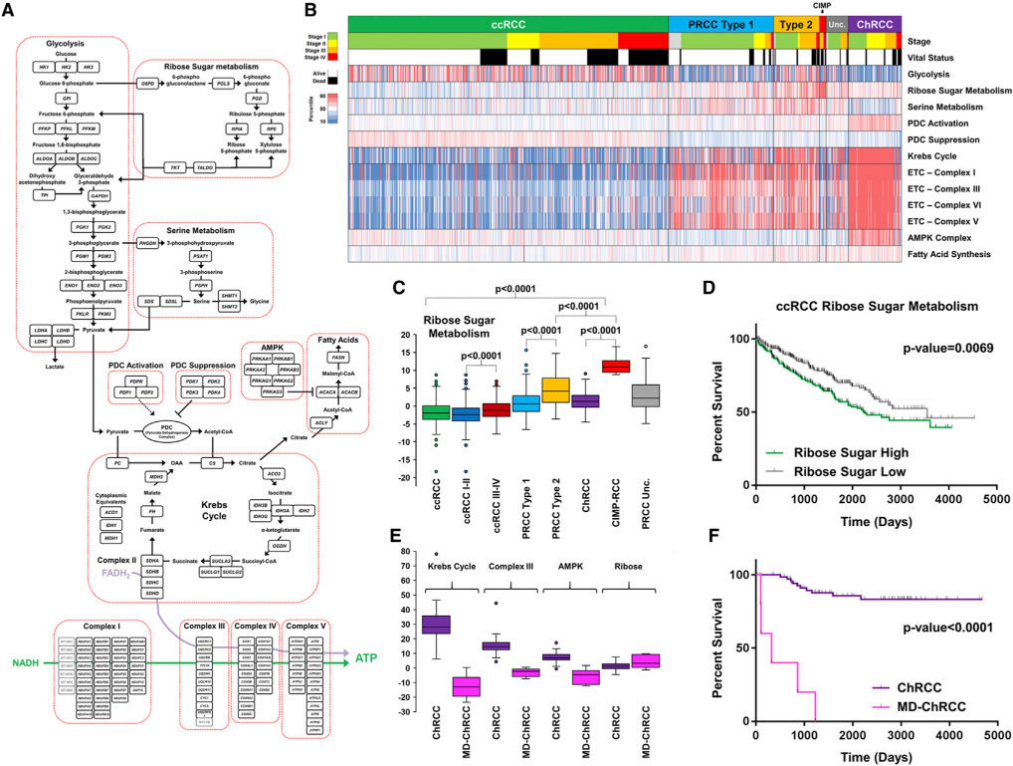
Metabolic Analysis of RCC Histologic Subtypes (A) Schematic of metabolic pathway genes selected for metabolic analysis. (B) Heatmap representation of the comparison of mRNA expression signatures for the selected metabolic processes between the different histologic RCC subtypes (ccRCC, green; type 1 PRCC, light blue; type 2 PRCC, orange; Unc. PRCC, gray; CIMP-RCC, red; ChRCC, purple). Tumor stage (stage I, light green; stage II, yellow; stage III, orange; stage IV, red) and vital status (alive, white; deceased, black) are indicated above the heatmap. (C) Comparative expression of the ribose sugar metabolism signature between the different histologic RCC (ccRCC, green; ccRCC stage I/II, dark blue; ccRCC stage III/IV, dark red; type 1 PRCC, light blue; type 2 PRCC, orange; Unc. PRCC, gray; CIMP-RCC, red; ChRCC, purple). (D) Differences in patient overall survival within ccRCC dependent upon expression of the ribose sugar metabolism signature (log-rank p value). (E) Comparative expression of the Krebs cycle, ETC Complex III, AMPK, and ribose sugar metabolism gene signatures between ChRCC and metabolically divergent (MD) ChRCC (ChRCC, purple; MD-ChRCC, pink). (F) Differences in patient overall survival between ChRCC and MD-ChRCC (log-rank p value).

Evaluation of tumor metabolism was performed by comparing the expression profiles for 12 major metabolic processes across all samples (Figure 5A and Table S3). Expression of the Krebs cycle and the electron transport chain (ETC) genes provided a clear distinction between the major histologic subtypes, with low expression in ccRCC and CIMP-RCC, high expression in ChRCC, and intermediate expression in type 1 and type 2 PRCC (Figure 5B). This correlated with increased expression of the pyruvate dehydrogenase complex (PDC) activation genes in ChRCC, that would help fuel the Krebs cycle and oxidative phosphorylation, and the increased expression of PDC suppression genes in ccRCC, which would result in glycolysis-dependent energy production (Figures 5B and S4A). Subtype analysis revealed that glycolytic gene expression was consistently higher in ccRCC and type 2 PRCC, while expression of the Krebs cycle genes was significantly higher in type 2 PRCC compared to type 1 PRCC (p < 0.0001) (Figure S4A). Although expression of PDC activation genes was low in all ccRCC, stage III-IV ccRCC demonstrated significantly lower expression than stage I-II ccRCC (p = 0.0005) and lower PDC activation gene expression in ccRCC was associated with decreased survival (p < 0.0001) (Figures S4A and S4B). Expression of 5′ AMP-activated protein kinase (AMPK), which negatively regulates fatty acid synthesis and positively regulates mitochondria production, was increased in ChRCC and decreased in the CIMP-RCC (Figure 5B). As previously observed in the TCGA ccRCC analysis, AMPK expression was significantly lower in stage III-IV ccRCC compared to stage I-II ccRCC (p = 0.0005), and lower expression correlated with poorer survival (p = 0.0005) (Figures S4A and S4B). Ribose sugar metabolism gene expression was increased in type 2 PRCC compared to type 1 PRCC (p < 0.0001) and greatly increased in CIMP-RCC in comparison to all other RCC subtypes (p < 0.0001) (Figure 5C). The increased ribose sugar metabolism expression previously associated with higher stage and poorer survival prognosis in ccRCC was confirmed in the current study (p = 0.0069), and increased ribose sugar metabolism expression was found to also be associated with decreased survival in PRCC (p = 0.0031) (Figures 5D and S4B).

Six ChRCC were identified that presented as distinct metabolic outliers within that histologic subtype (Figure S5A). Compared to the other ChRCC, these samples had low expression of the Krebs cycle and electron transport chain genes, lower expression of the AMPK pathway genes, and increased expression of the genes in the ribose synthesis pathway, and all these features were associated with poorer prognosis in other RCC histologic subtypes (Figure 5E). These metabolically divergent (MD) ChRCC were high stage (stage III or IV), demonstrated the hypermethylation pattern described above, lacked the chromosomal copy number losses normally associated with ChRCC, and were associated with much poorer survival in comparison to other ChRCC (p < 0.0001) (Figures 5F and S5A). Four of the six MD-ChRCC were found to have sarcomatoid de-differentiation (Figure S5B). Several of these MD-ChRCCs were initially misidentified as ccRCC and then re-assigned after a pathology review by urologic pathology experts, reflecting their unusual pathology.

### Immune Signature Analysis

**Figure 6:**
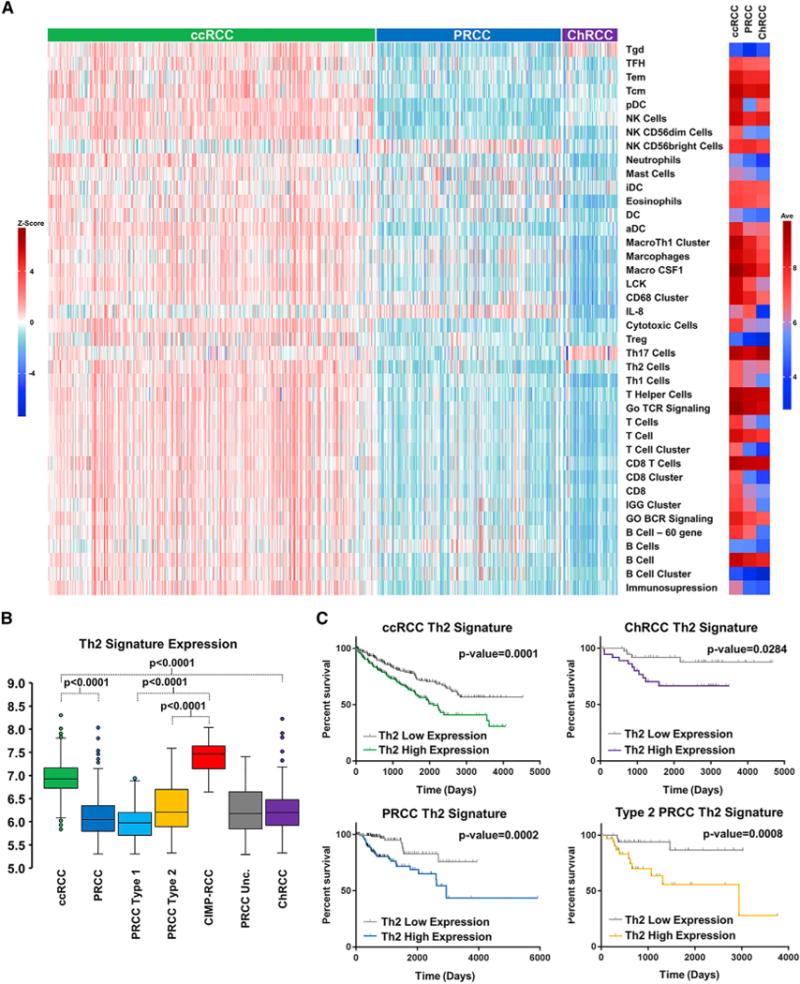
Immune Signature Analysis (A) Supervised clustering of immune gene signature (IGS) expression by individual sample (left) or mean IGS expression (right) for the different histologic RCC subtypes (ccRCC, green; PRCC, blue; ChRCC, purple). (B) Comparative expression of the Th2 gene signature between the histologic RCC subtypes (ccRCC, green; PRCC, blue; type 1 PRCC, light blue; type 2 PRCC, orange; CIMP-RCC, red; unclassified PRCC, gray; ChRCC, purple) (t test). (C) Comparative differences in patient overall survival within the histologic RCC subtypes (ccRCC, green; PRCC, blue; type 2 PRCC, orange; ChRCC, purple) dependent upon the Th2 gene signature (log-rank p value).

An increased immune cell infiltrate gene expression signature in ccRCC in comparison to PRCC and ChRCC has been elucidated by several studies, including importance of single gene markers such as *PDCD1* (PD1) and *CD247* (PDL1) (Chen et al., 2016; Geissler et al., 2015). Analysis using a refined immune cell gene-specific signatures (Table S4) confirmed that, with the exception of the Th17, IL-8, and CD56^bright^ NK cell gene signatures, there was nearly universal upregulation of these immune signatures in ccRCC compared to the PRCC or ChRCC (Figures 6A and S6A). The T helper 17 cell (Th17) gene signature had increased expression in ChRCC, while the IL-8 and CD56^bright^ NK cell gene signatures had increased expression in PRCC. Separation of the PRCC tumors highlighted distinct differences in the CIMP-RCC compared to the remaining PRCC, including increased expression of the Th2, activated dendritic cell (aDC), plasmacytoid dendritic cell (pDC), and Mast cell gene signatures, that produced a profile more similar to ccRCC (Figures 6B and S6B). T cell receptor (TCR) profiling used to identify TCR clonotype expression within the cohort demonstrated patterns of subtype-specific TCR clonotype expression suggesting variation in T cell response between ccRCC, PRCC, and ChRCC tumors (Figure S6C). In accordance with previous findings, gene signatures correlated with reduced survival, including signatures that represented T cells, B cells, macrophages, dendritic cells, and NK cells (Figure S6D). The T helper 2 cell (Th2) gene signature was increased in most ccRCC, all CIMP-RCC, and in outliers of the ChRCC, with six of the top seven Th2 gene signature scores within the ChRCC tumors representing the aggressive MD-ChRCC tumors (Figure 6B). Notably, an increased Th2 gene signature represented the only biomarker that correlated with poor survival when evaluated within each major histologic subtype, ccRCC (p = 0.0001), PRCC (p = 0.0002), and ChRCC (p = 0.0284) (Figure 6C). Subtype separation of the PRCC demonstrated that this correlation was present only in PRCC type 2 (p = 0.0089) (Figure 6C). Expression of the Th17 gene signature was associated with increased survival in ccRCC (p = 0.0021), with additional positive correlation in ChRCC (p = 0.0362) (Figure S6E).

## DISCUSSION

The importance of identifying and differentiating the subtypes and even rare variants of renal cell carcinoma (RCC) is critical for management and treatment of patients affected with this disease. Although histologic subtyping divides tumors into distinct RCC groups, it is limited in its ability to provide in-depth analysis of mechanisms that produce these differences. In the present study, comprehensive genetic and genomic analysis demonstrated that different histologically defined RCC subtypes are characterized by distinctive mutations, chromosomal copy number alterations, and expression patterns of mRNA, miRNA, and lncRNA, and that the combination of histology plus genomics provides unique insight into patient-centered management. These combined differentiating features, obtained via a tumor or liquid biopsy, provide invaluable information and prognostic biomarkers to guide clinical and surgical management.

While this study characterizes the differences between the major RCC histologic subtypes, shared features within the RCC subtypes may also provide more universal prognostic markers and targets for therapy. The loss of *CDKN2A*, which encodes p16, by either gene deletion, promoter hypermethylation, or mutation, found in 16% of RCC, correlated with poor survival in ccRCC, PRCC, and ChRCC. Loss of *CDKN2A* is known to correlate with poor outcome in ccRCC, PRCC, and other cancer types, but this demonstrates that it is a universal feature of RCC and is potentially targetable with CDK4/6 inhibitors that target the downstream effects of p16 loss (Hamilton and Infante, 2016). Increased promoter hypermethylation also was found to be associated with decreased survival in ccRCC, PRCC, and ChRCC. Previous studies have shown increased levels of DNA hypermethylation correlating with poorer outcome that was limited to ccRCC and PRCC without identifying potentially impacted pathways (Cancer Genome Atlas Research Network, 2013; Cancer Genome Atlas Research Network et al., 2016; Chen et al., 2016). This study highlighted hypermethylation of WNT pathway regulatory genes and demonstrated that analysis of hypermethylation in two specific WNT regulatory genes, *SFRP1* and *DKK1*, recapitulated the correlation with decreased survival in ccRCC, PRCC, and ChRCC. Increased DNA methylation was associated with *SETD2* mutation, which is known to alter DNA methylation patterns (Tiedemann et al., 2016), in ccRCC and PRCC, and increased DNA methylation was similarly associated with *PBRM1* mutation in PRCC. Hypermethylation of *SFRP1* and *DKK1* could provide a prognostic biomarker for RCC and has been previously proposed in ccRCC (Hirata et al., 2011; Ricketts et al., 2014; Urakami et al., 2006). This suggests that treatment with de-methylating agents could be beneficial in patients with increased levels of promoter hypermethylation.

This study also demonstrated features that were shared by some RCC subtypes, but not all, and underlines the importance of evaluating these alterations within each RCC subtype as well as across all subtypes. Previous studies using TCGA data and other cohorts have shown that *BAP1* mutation, but not *PBRM1* mutation, correlates with poor survival in ccRCC and these correlations were confirmed in a mixed cohort of ccRCC and PRCC TCGA tumors (Chen et al., 2016; Hakimi et al., 2013; Kapur et al., 2013). By analysis of the histologic subtype of RCC, we confirmed these correlations in ccRCC and showed that while *BAP1* mutations did not correlate with survival in PRCC, *PBRM1* mutations did associate with poor survival in type 1 PRCC.

Assessment of the RCC metabolic states within RCC revealed significant metabolic alterations. High ribose metabolism gene expression was present in both ccRCC and CIMP-RCC, with CIMP-RCC showing the greatest expression, likely due to the increased production of NADPH counteracting the cellular stress induced by the loss of fumarate hydratase in these tumors (Ooi et al., 2011; Patra and Hay, 2014; Sourbier et al., 2014). Type 2 PRCC had increased expression of the glycolysis, ribose metabolism, and Krebs cycle genes in comparison to type 1 PRCC, suggesting a more metabolically active tumor, consistent with its more aggressive nature. Increased expression of the ribose metabolism genes correlated with poor survival in both ccRCC and PRCC. These findings suggest that targeting the ribose metabolism pathway could be a potential therapeutic approach in ccRCC, type 2 PRCC, and CIMP-RCC.

The immune expression signature is an increasingly important feature of ccRCC, given the recent introduction of checkpoint inhibitor therapy (Lee and Motzer, 2016; Motzer et al., 2015), and patterns of immune infiltration in RCC have been observed in several studies (Chen et al., 2016; Geissler et al., 2015). The role of this feature in determining the therapeutic responsiveness of ccRCC will be important in future therapeutic planning. A recent study using TCGA RCC data demonstrated that differences in expression in specific checkpoint-related genes, such as *PDCD1* (PD1) and *CD247* (PDL1), correlated with patient survival within ccRCC cases (Chen et al., 2016). While we observed the same general pattern as previously seen with PRCC overall demonstrating little expression of the immune signature associated with ccRCC, we found CIMP-RCC to have an increased immune signature expression for select immune gene signatures, including the Th2 gene signature, like that seen in ccRCC. This suggests this most aggressive type of RCC, CIMP-RCC, may benefit from checkpoint inhibitor therapy in a similar manner to ccRCC. Although the Th2 gene signature was considerably higher in ccRCC and CIMP-RCC tumors compared to other tumor subtypes, the relative levels of Th2 gene signature within each major RCC histologic subtype correlated with poor patient survival, as had been previously observed in ccRCC (Şenbabaoǧlu et al., 2016). This suggests that once expression ranges are defined for each subtype, this Th2 gene signature could provide a useful prognostic marker for all RCC subtypes.

While the current study confirmed the previous finding of CIMP-RCC as a specific PRCC subtype, in this analysis we identified a subset of metabolically divergent (MD) ChRCC that also demonstrated a uniform and distinct metabolic expression pattern associated with extremely poor survival. The MD-ChRCC had decreased Krebs cycle, ETC, and AMPK gene expression and increased ribose metabolism gene expression similar to higher-stage ccRCCs. All the MD-ChRCC were high stage and generally lacked the classic ChRCC-associated pattern of chromosomal loss, and most demonstrated sarcomatoid differentiation. A recent study has also shown a correlation between the absence of the classical ChRCC chromosome loss and aggressive, high-grade, metastatic ChRCC (Casuscelli et al., 2017). Many of these MD-ChRCC features are represented in a recently characterized sarcomatoid ChRCC-derived cell line that could provide a model for further investigation of these tumors (Yang et al., 2017). The combination of histopathology and expression analysis may provide a definitive classification for ChRCC and enable the identification of aggressive variants that may require alternative management and therapy, including the potential for adjuvant therapy.

Understanding the molecular and genetic features that characterize the RCC subtypes will provide the foundation for the development of improved methods for both clinical and surgical management and therapies to treat this disease. Besides identifying discrete genomic characteristics that are critical for the understanding of individual RCC subtypes, we have identified unifying features, such as the effect of the Th2 immune gene signature on survival, which cross disease subtypes and which will help provide the foundation for the development of effective forms of therapy for patients with advanced disease.

## STAR★METHODS

### KEY RESOURCES TABLE

**Table:** 

REAGENT or RESOURCE	SOURCE	IDENTIFIER
Biological samples		
Primary tumor samples	Multiple tissue source sites, processed through the Biospecimen Core Resource	See Biospecimen Acquisition in EXPERIMENTAL MODEL AND SUBJECT DETAILS
Critical Commercial Assays		
Genome-Wide Human SNP Array 6.0	ThermoFisher Scientific	Cat: 901153
Infinium HumanMethylation450 BeadChip Kit	Illumina	Cat: WG-314-1002
Illumina Barcoded Paired-End Library Illumina Preparation Kit	Illumina	https://www.illumina.com/techniques/sequencing/ngs-library-prep.html
TruSeq RNA Library Prep Kit	Illumina	Cat: RS-122-2001
TruSeq PE Cluster Generation Kit	Illumina	Cat: PE-401-3001
Deposited Data		
Raw and processed clinical, array and Genomic sequence data.	Data Commons	https://portal.gdc.cancer.gov/legacy-archive
Processed RNA sequence data	Gene Expression Omnibus	https://www.ncbi.nlm.nih.gov/geo/
Digital pathology images	Cancer Digital Slide Archive	http://cancer.digitalslidearchive.net/
Software and Algorithms		
ConsensusClusterPlus	Wilkerson and Hayes, 2010	http://bioconductor.org/packages/release/bioc/html/ConsensusClusterPlus.html
Cufflinks	Trapnell et al., 2013	https://cole-trapnell-lab.github.io/cufflinks/
DESeq2 package	Love et al., 2014	https://bioconductor.org/packages/release/bioc/html/DESeq2.html
Genome Analysis Toolkit	McKenna et al., 2010	https://software.broadinstitute.org/gatk/
GSNAP	Wu and Watanabe, 2005	http://research-pub.gene.com/gmap/
MiXCR v1.7.1	Bolotin et al., 2013	https://mixcr.readthedocs.io/en/latest/
MuTect	Cibulskis et al., 2013	http://archive.broadinstitute.org/cancer/cga/mutect
MUSE	Fan et al., 2016	http://bioinformatics.mdanderson.org/main/MuSE
Pindel	Ye et al., 2009	http://gmt.genome.wustl.edu/packages/pindel/index.html
MUSCLEt	Edgar, 2004	http://www.drive5.com/muscle/
MtoolBox	Calabrese et al., 2014	https://sourceforge.net/projects/mtoolbox/
Radia	Radenbaugh et al., 2014	https://github.com/aradenbaugh/radia
samr	Li and Tibshirani, 2013	https://cran.r-project.org/web/packages/samr
Samtools	Li et al., 2009	http://samtools.sourceforge.net/
Somatic Sniper	Larson et al., 2012	http://gmt.genome.wustl.edu/packages/somatic-sniper/
STAR	Dobin et al., 2013	https://github.com/alexdobin/STAR
VarScan2	Koboldt et al., 2012	http://varscan.sourceforge.net/
WGCNA package	Langfelder and Horvath, 2008	https://labs.genetics.ucla.edu/horvath/CoexpressionNetwork/Rpackages/WGCNA/
Other		
Firehose, FireBrowse	The Broad Institute, Cambridge MA	https://gdac.broadinstitute.org/, http://firebrowse.org/

### CONTACT FOR REAGENT AND RESOURCE SHARING

Further information and requests for resources and reagents should be directed to and will be fulfilled by the Lead Contact, Dr. W. Marston Linehan (WML@nih.gov).

### EXPERIMENTAL MODEL AND SUBJECT DETAILS

#### Biospecimen Acquisition

All biospecimens were acquired by the Cancer Genome Atlas (TCGA) Resource Network. Surgically resected tumor specimens were collected from patients diagnosed with renal cell carcinoma (RCC) that had preferably not received any prior treatment for their disease, such as chemotherapy or radiotherapy. Individual institutional review boards at each tissue source site reviewed the protocols and consent documentation and approved the submission of cases to TCGA. All tumors were staged per the American Joint Committee on Cancer (AJCC) and each primary tumor specimen had a matched normal tissue specimen. The tissue source sites for the Cancer Genome Atlas Research Network are listed in the Cancer Genome Atlas Research Network author list for this project.

The initial 894 samples of kidney cancer that were submitted to TCGA were re-evaluated by a panel of expert pathologists that excluded several samples due to inconsistent or incorrect histologic classification or therapy prior to sample collection. This accounts for the variation in samples compared to the previous Chen et al. study (Chen et al., 2016). The approved 843 tumors were subdivided by histologic subtype into 6 groups consisting of 488 clear cell (cc)RCC, 160 Type 1 papillary (P)RCC, 70 Type 2 PRCC, 34 unclassified PRCC, 10 CpG island methylator phenotype-associated (CIMP-)RCC, and 81 chromophobe (Ch)RCC based on the original pathology reports or re-evaluation by a panel of expert urologic pathologists. Six hundred and ninety-three of the tumors had been analyzed in the three individual TCGA marker papers. The clinical and genetic characteristics of these patients are described in Table S1 in the Supplementary Appendix.

### METHOD DETAILS

#### Somatic Exome Mutation Analysis

Somatic exome sequencing data was available and downloaded for 804 of the 843 pan-kidney tumors representing 463 ccRCC, 266 PRCC, 74 ChRCC. The tumors with sequencing data are designated within Table S1 and all data is accessible via the NCI genome data commons (https://gdc.cancer.gov/).

A combined MAF (Mutation Annotation Format) file for all samples was produced by extracting the relevant sample data from the TCGA unified ensemble “MC3” call set and supplementing this with data from the original three TCGA KIRC, KICH, and KIRP publication for samples not present in the TCGA MC3 dataset. The TCGA unified ensemble “Multi-Center Mutation Calling in Multiple Cancers” (“MC3′”) call set is the public, open-access, dataset of somatic mutation calls (SNVs and indels) produced as part of the capstone project using all available of cases within TCGA using six different algorithms (MuTect, MuSE, Pindel, Somatic Sniper, VarScan2 and Radia) from four centers (Cibulskis et al., 2013; Fan et al., 2016; Koboldt et al., 2012; Larson et al., 2012; Radenbaugh et al., 2014; Ye et al., 2009).

The significantly mutated genes (SMGs) that had been previously identified by the MutSigCV algorithm in the previous TCGA KIRC, KICH, and KIRP publications were used as the reference SMGs when evaluating the entire pan-kidney dataset. Pathway analysis for the HIF pathway, HIPPO pathway, NRF2/ARE pathway, PI3K/AKT pathway and the chromatin remodeling pathways was performed using gene lists described in Table S2. The pathway analysis involving genes known to be activated in cancer, such as *MTOR*, *PIK3CA*, and *NFE2L2*, were limited to missense mutations only.

#### SNP Array-Based Copy Number Analysis

The gene level copy number data (focal_data_by_genes) generated by Affymetrix SNP 6.0 arrays using protocols at the Genome Analysis Platform of the Broad Institute (McCarroll et al., 2008) was available for 832 of the 843 pan-kidney tumors representing 481 ccRCC, 271 PRCC, and 80 ChRCC. Tumors with copy number data are designated within Table S1 and all data is accessible via the NCI genome data commons (https://gdc.cancer.gov/). Estimates for gross chromosomal arm gain or loss were produced by averaging the copy number values for all genes within each region. Average values greater than 0.3 were considered chromosomal gain and average values less than −0.3 were considered chromosomal loss. For individual gene copy number analysis, such as *CDKN2A* loss, copy number values of less than −0.4 were considered to represent deletion.

#### RNA Expression Data Analysis

The level 3 RNA-Seq upper quartile normalized RSEM data was available for 839 of the 843 pan-kidney tumors representing 485 ccRCC, 273 PRCC, and 81 ChRCC. Tumors with RNA-seq data are designated within Table S1 and all data is accessible via the NCI genome data commons and the Gene Expression Omnibus (https://gdc.cancer.gov/ and https://www.ncbi.nlm.nih.gov/geo/). Analysis of the RNA data was split into miRNA analysis, lncRNA analysis, mRNA signature analysis, and immune gene signature analysis.

#### mRNA Signature Analysis

Raw count data for each sample included was obtained from Gene Expression Omnibus (GSE62944) (Rahman et al., 2015). All subsequent analyses were performed in R open source programming language. For differential expression analysis, RPKM values were calculated from RNaseq raw counts and upper quantile normalized. For hierarchical clustering and WGCNA, raw count data were processed and normalized using the variance stabilizing transformation (VST) algorithm implemented by the DESeq2 package (Love et al., 2014).

Scale-free weighted signed gene co-expression networks were constructed by the WGCNA package (Langfelder and Horvath, 2008). Using the top 11000 varying genes according to their standard deviation, WGCNA was restricted to the 9000 most connected genes. First, a pairwise gene correlation matrix was calculated with a Pearson correlation analysis, which was transformed into a weighted matrix to produce an adjacency matrix after raising values by an exponent beta (β = 16). Then the adjacency was transformed into a topological overlap matrix (TOM). The dynamic tree cut method was used for module identification from the hierarchical clustering of genes using 1-TOM as the distance measure with a deepSplit value of 2 and a minimum size cutoff of 50 genes. Highly similar modules were identified by clustering and then merged together with a height cut-off of 0.2. Finally, modules and their relationship to clinical traits were identified using Pearson correlation analysis between the modules and external traits. Functional annotation of identified modules was performed using tools provided by the WGCNA package.

Kmeans consensus clustering was performed using ConsensusClusterPlus package (Wilkerson and Hayes, 2010). The K-value of 6 was selected according to the consensus cumulative distribution function, where K > 6 did not produce any appreciable increase in consensus (Monti et al., 2003; Wilkerson and Hayes, 2010). Hierarchical unsupervised cluster analysis was performed using 7738 genes pertaining to selected WGCNA modules (see Figure 4 for modules). Hierarchical clustering was performed using average linkage of Euclidean distance.

#### Non-coding RNA (lncRNA and miRNA) Sequencing and Analysis

mRNA sequence reads were aligned to the human reference genome (hg38) and transcriptome (Ensembl v82, September 2015) using STAR 2.4.2a (Dobin et al., 2013). STAR was run with the following parameters: minimum/maximum intron sizes were set to 30 and 500,000, respectively; noncanonical, unannotated junctions were removed; maximum tolerated mismatches was set to 10; and the outSAMstrandField intron motif option was enabled. The Cuffquant command included with Cufflinks 2.2.1 (Trapnell et al., 2013) was used to quantify the read abundances per sample, with fragment bias correction and multiread correction enabled, and all other options set to default. To calculate normalized abundance as fragments per kilobase of exon per million fragments mapped (FPKM), the Cuffnorm command was used with default parameters. From the FPKM matrix for the 80 tumor samples, we extracted 8167 genes with “lincRNA” and “processed_transcript” Ensembl biotypes.

From the matrix of 8167 lncRNAs (above), we extracted FPKM profiles for 499 lncRNAs that were robustly expressed (mean FPKM ≥ 1) and highly variable (≥ 92.5th FPKM variance percentile) across the n = 833 primary tumor cohort. We identified groups of samples with similar expression profiles by unsupervised consensus clustering with *ConsensusClusterPlus* (CCP) 1.20.0 (Wilkerson and Hayes, 2010). Calculations were performed using Pearson correlations, partitioning around medoids (PAM), a gene fraction of 0.95, and 200 iterations. It was anticipated that a hierarchically-related series of finer-grained and coarser-grained sets of subtypes may be available from a clustering analysis, that a particular clustering solution (i.e., number of subtypes) from such a series may be a more informative choice for a particular question and context, and that results from multiple data types may need to be considered in order to identify a clustering solution to report on because it is effective in contributing to the overall insights (Aine et al., 2015; Ronan et al., 2016). A consensus clustering solution for lncRNAs was selected by initially considering information for different numbers of clusters and for a range of clustering approaches. The reported clustering solution considered four main factors: a) the consensus membership heatmaps and dendrograms; b) the ‘delta’ plot showing how the area under the cumulative distribution function of consensus membership values increases as the numbers of clusters increases; c) the profile of silhouette width calculated from the consensus memberships, which we take as a measure of typical versus atypical cluster membership; and d) how KIRC, KIRP Type 1 and 2, and KICH samples were separated and subdivided by the clusters. Thus, we selected an 8-cluster solution after assessing consensus membership heatmaps, dendrograms, and CCP clustering metrics for up to 10 clusters. To visualize typical versus atypical cluster members, we used the R *cluster* package to calculate a profile of silhouette widths (W_cm_) from the consensus membership matrix. To generate an abundance heatmap for the 8-cluster result, used the *pheatmap* R package (v1.0.2). We ordered the columns to correspond to the above consensus clustering result. We manually transferred the upper dendrogram graphic from the consensus result to the heatmap graphic that we were generating. For the rows, we identified a subset of lncRNAs that had a mean FPKM ≥ 10 and a SAM multiclass (samr 2.0) (Li and Tibshirani, 2013) q value of 0.0 across the clusters (see differential abundance, below), transformed the FPKM matrix by log_10_(FPKM + 1), then, in *pheatmap*, scaled the rows and clustered them with a Pearson distance metric and Ward clustering.

We compared unsupervised clusters to clinical and molecular covariates by calculating contingency table association p values using R, with a Chi-square or Fisher exact test for categorical data, and a Kruskal-Wallis test for real-valued data.

We generated miRNA sequencing (miRNA-seq) data from messenger RNA-depleted RNA, as describe in (Chu et al., 2016). Briefly, we aligned ~22-nt reads to the GRCh37/hg19 reference human genome, assigned read count abundances to miRBase v16 stem-loops and 5p and 3p mature strands, and assigned miRBase v20 mature strand names to MIMAT accession IDs. Note that while we used only reads with exact-match alignments in calculating miRNA abundances, BAM files available from the Genomics Data Commons (https://gdc.cancer.gov/) include all sequence reads.

For miRNA, mature strand (miR) sequencing data for n = 811 primary tumors, we extracted normalized abundance (RPM) data matrices for ccRCC (n = 457), PRCC (n = 274), and ChRCC (n = 80, which included n = 65 KICH and n = 15 that were originally part of the KIRC cohort). From RPM data matrices for the 457, 274 and 65 original samples respectively, we identified the 304 miRs that were the most-variant 25% (of 1214 miRBase v16 strands) for each cohort. Combining the three lists gave 369 unique miR names. In a batch-corrected data matrix containing 743 miRs and 9,555 primary tumor samples (of 10,825 total samples), 367 of the 369 miRs were available, and we generated a batch-corrected data matrix with 367 miR and 811 primary tumor samples that was the input to unsupervised clustering.

Using ConsensusClusterPlus v1.40.0 we assessed consensus membership heatmaps and other metrics for six approaches, using Pearson or Spearman correlations as distance metrics, and hierarchical, partitioning around meoids (PAM) or k-means clustering. For each approach, we assessed solutions with between two and nine clusters. We report on a 6-cluster solution for Spearman correlations, PAM clustering, and 1000 iterations with a random mature-strand fraction of 0.85 for each iteration. We used a similar selection methodology for the 6-cluster solution as was described above for the lncRNAs.

We used an approach similar to that described above for lncRNAs to generate a clustering heatmap for miRNAs. We first identified miRNAs that were differentially abundant between the unsupervised miRNA clusters using a SAM multiclass analysis (samr 2.0) (Li and Tibshirani, 2013) in R, with the 367-×-811 RPM input data matrix, 1000 permutations, no array centering, a Wilcoxon test statistic, and an FDR threshold of 0.05. For the heatmap we used miRNAs that had larger SAMseq scores and q-values of 0.0. We ordered the data matrix columns to match the clustering result, manually transferred over the upper dendrogram from the consensus clustering graphic, then transformed each row of the matrix by log_10_(RPM+1) and used the *pheatmap* R package (v1.0.2) to scale and cluster only the rows.

We generated a Kaplan-Meier plot for the miRNA clusters using the R survival package v2-41.3. We compared unsupervised clusters to clinical and molecular covariates by calculating contingency table association p values using R, with a Chi-square or Fisher exact test for categorical data, and a Kruskal-Wallis test for real-valued data.

#### Immune Gene Signature Analysis

Immune gene signatures were derived from previously published works (Beck et al., 2009; Bindea et al., 2013; Fan et al., 2011; Iglesia et al., 2014; Kardos et al., 2016; Palmer et al., 2006; Rody et al., 2009; Rody et al., 2011; Schmidt et al., 2008; Teschendorff et al., 2007). RSEM upper quartile normalized, log-2 transformed, and mean centered RNA-seq data was matched to predefined immune gene signature clusters via Entrez IDs. Each gene signature was calculated as the average value of all genes included in the signature (Table S4). Differential expression for each gene signature was analyzed between kidney cancer types and subtypes via one-way ANOVA. These p values were adjusted for multiple testing using the Benjamini-Hochberg procedure. For hazard ratio forest plots, univariate Cox proportional hazards (CoxPH) model was used with signature/clinical variable as a continuous variable compared to patient overall survival. T cell receptor repertoire analysis was performed using MiXCR v1.7.1 on default alignment and assemble settings (Bolotin et al., 2013). Diversity measurements were analyzed between kidney cancer types and subtypes via Mann-Whitney U-test.

#### DNA Methylation Analysis

Two generations of Illumina Infinium DNA Methylation BeadArrays, including the HumanMethylation27 (HM27) and HumanMethylation450 (HM450) arrays, were used to assay 824 pan-kidney tumors (65 KICH, 485 KIRC and 274 KIRP) and 392 normal kidney samples in total (Table S1). All data is available from the NCI genome data commons (https://gdc.cancer.gov/).

Data from HM27 and HM450 were combined and further normalized using a probe-by-probe proportional rescaling method to yield a common set of 22,601 probes with comparative methylation levels between the two platforms, as described in details on Synapse (Syn7073804). Briefly, we rescaled data on HM27 based on between-platform difference measured by technical replicates. Probes were further filtered based on 34 technical replicates measured together with the KIRC samples by removing those showing a standard deviation of 0.05 or above. Unsupervised clustering was performed based on cancer-specific autosomal loci, which were defined as unmethylated probes in all normal tissue types as well as sorted blood populations (mean beta value < 0.2), but methylated (beta value > 0.3) in more than 5% samples within any of the kidney tumor type (for tumor type with less than 100 samples, we require the portion of methylated samples to be greater than 10% instead). To minimize the influence of tumor purity, we dichotomize the methylation data into 0’s and 1’s with a beta value cut off of 0.3, and used Ward’s method to cluster the distance matrix computed with the Jaccard Index. Heatmaps were generated based on row and column orders calculated as above and colored by dichotomized beta values.

The DNA methylation level as interrogated by cg07684796, cg15839448 was used for DKK1, and SFRP1, respectively, with a beta value of 0.3 or more considered evidence for epigenetic silencing.

#### Survival Analysis

The Kaplan-Meier method was used to generate curves for overall survival and the Log-rank test was used to assess the univariate survival differences with no correction for multiple testing, unless otherwise stated in specific analyses. Overall survival was defined as the time from the nephrectomy to death of any cause.

#### mtDNA Sequence and Copy Number Analysis

Whole exome sequencing (WXS) BAM files, sequenced at BCM Sequencing Center, were obtained for 66 ChRCC, 153 ccRCC, and 128 PRCC tumor samples and corresponding blood or normal tissue DNA. BAM files were used as input of the MToolBox pipeline, that includes GSNAP, MUSCLE, and SAMtools, to align reads to the Revised Cambridge Reference Sequence (rCRS) for human mitochondrial DNA, extract variant alleles, quantify their heteroplasmy levels and related confidence intervals, and obtain functional annotation of the identified variants.(Calabrese et al., 2014; Edgar, 2004; Li et al., 2009; Wu and Watanabe, 2005) Samples with > 75% mtDNA sequence coverage in Tumor and Normal DNA and variants with > 5% mutation load were considered for further analysis (61 ChRCC, 66 ccRCC, and 99 PRCC). Variant tables from tumor and corresponding normal DNA were compared to determine somatic mutations, which were then classified according to criteria outlined in Figure S2F.

The mtDNA copy number (m) was calculated for samples with mtDNA sequence data as the ratio of the number of sequencing reads aligning to the mitochondrial genome (r_m_) and the nuclear genome (r_n_) according to the following formula: m = r_m_/r_n_ × R. Correction for tumor ploidy and purity (R) was calculated as RTumor = (Purity × Ploidy+(1 Purity) × 2)/2. Allele-specific copy number and estimates of tumor ploidy and purity were calculated with ASCAT (Reznik et al., 2016; Reznik et al., 2017; Van Loo et al., 2010) using matched Affymetrix SNP6 array data from tumor and normal tissue. Batch effect on exome enrichment was corrected for by applying a linear model that accounted for plate and center IDs as well as tissue type.

### QUANTIFICATION AND STATISTICAL ANALYSIS

For all analyses, significance was determined as a p value < 0.05 and corrected for multiple testing where specified. Univariate analysis was performed unless otherwise specified. Survival analyses were performed using GraphPad Prismâ (GraphPad Software, Inc.) or by individually specified methodologies. In all cases the “n” represents individual patients from which a single tumor was evaluated.

### DATA AND SOFTWARE AVAILABILITY

Raw and processed clinical, array and sequence data are all available via the Genomic Data Commons download portal (https://portal.gdc.cancer.gov) or Gene Expression Omnibus (https://www.ncbi.nlm.nih.gov/geo/-GSE62944) and the digital pathology images are all available from the Cancer Digital Slide Archive (http://cancer.digitalslidearchive.net/)

## Supplementary Material

1

2

3

4

5

6

## Highlights

- ***BAP1***, ***PBRM1***, and metabolic pathway changes correlate with RCC subtype-specific survival
- DNA hypermethylation/***CDKN2A*** alterations associate with poor survival in all RCC subtypes
- Immune gene signatures increased in ccRCC and CIMP-RCC
- Increased Th2 gene signature within each RCC subtype associates with poorer survival
