# Supplementary material for: The Cancer Genome Atlas Comprehensive Molecular Characterization of Renal Cell Carcinoma
Source: Cell Rep. Author manuscript; Available in PMC 2018 Aug 3. (PMC6075733; doi:10.1016/j.celrep.2018.03.075)
Supplement: 1 [file NIHMS958988-supplement-1.pdf]

Cell Reports, Volume *23*

Supplemental Information

The Cancer Genome Atlas Comprehensive Molecular Characterization of Renal Cell Carcinoma

Christopher J. Ricketts, Aguirre A. De Cubas, Huihui Fan, Christof C. Smith, Martin Lang, Ed Reznik, Reanne Bowlby, Ewan A. Gibb, Rehan Akbani, Rameen Beroukhim, Donald P. Bottaro, Toni K. Choueiri, Richard A. Gibbs, Andrew K. Godwin, Scott Haake, A. Ari Hakimi, Elizabeth P. Henske, James J. Hsieh, Thai H. Ho, Rupa S. Kanchi, Bhavani Krishnan, David J. Kwaitkowski, Wembin Lui, Maria J. Merino, Gordon B. Mills, Jerome Myers, Michael L. Nickerson, Victor E. Reuter, Laura S. Schmidt, C. Simon Shelley, Hui Shen, Brian Shuch, Sabina Signoretti, Ramaprasad Srinivasan, Pheroze Tamboli, George Thomas, Benjamin G. Vincent, Cathy D. Vocke, David A. Wheeler, Lixing Yang, William T. Kim, A. Gordon Robertson, The Cancer Genome Atlas Research Network, Paul T. Spellman, W. Kimryn Rathmell, and W. Marston Linehan

Table of Contents

Supplemental Figures	Page
Figure S1 related to Figure 1: Cluster analysis of tumor mRNA, miRNA, and lncRNA	2
Figure S2 related to Figure 2: Somatic and Mitochondrial Mutation Analysis	6
Figure S3 related to Figure 3: Methylation Cluster Analysis	12
Figure S4 related to Figure 5: RCC Tumor Metabolic Analysis	14
Figure S5 related to Figure 5: ChRCC Metabolic Analysis	17
Figure S6 related to Figure 6: Immune Expression Profile Analysis	19

Figure S1A

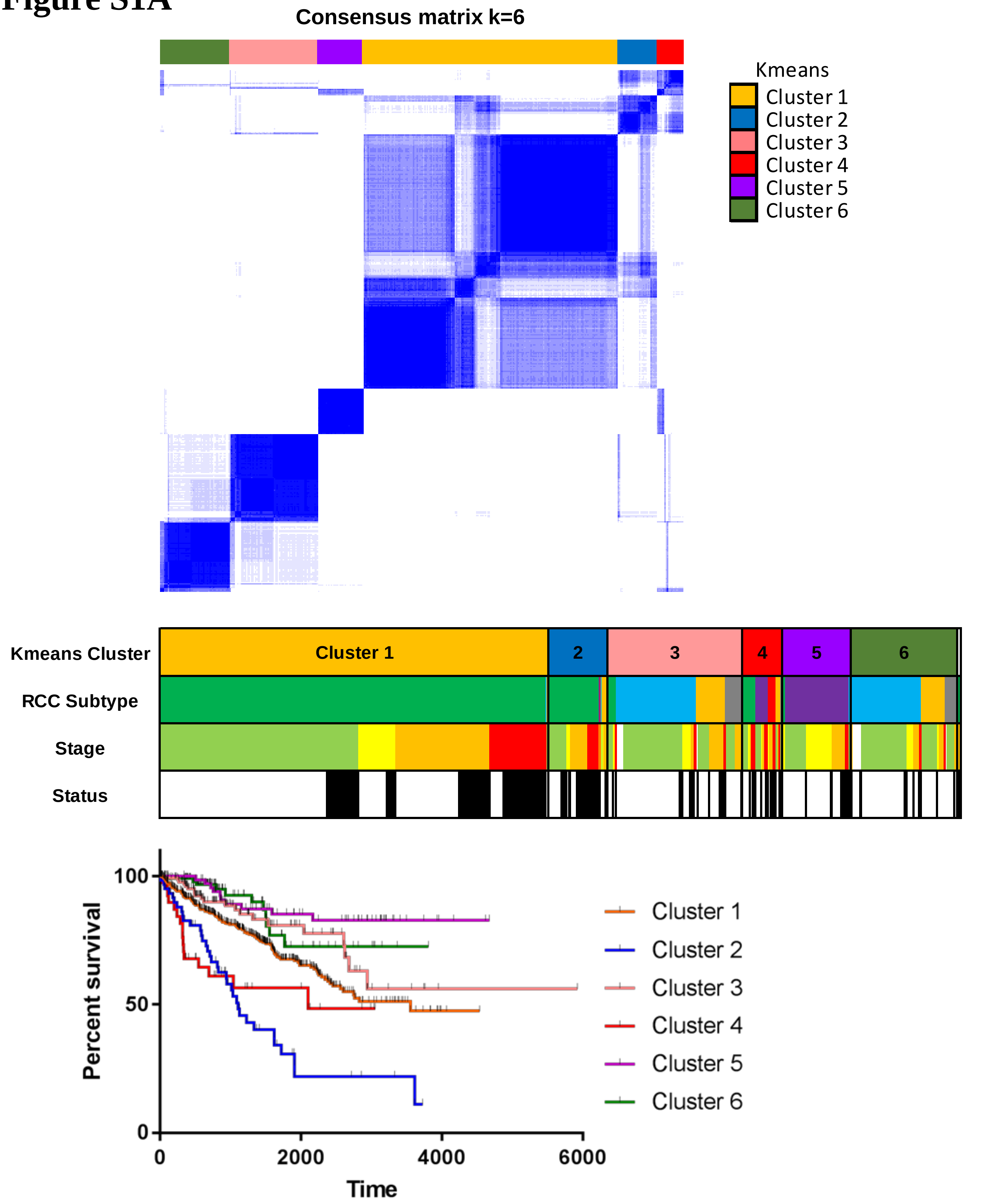

Cluster 1 vs. 2 – p-value<0.0001
Cluster 3 vs. 4 – p-value=0.0003
Cluster 6 vs. 4 – p-value<0.0001

Figure S1

Figure S1B

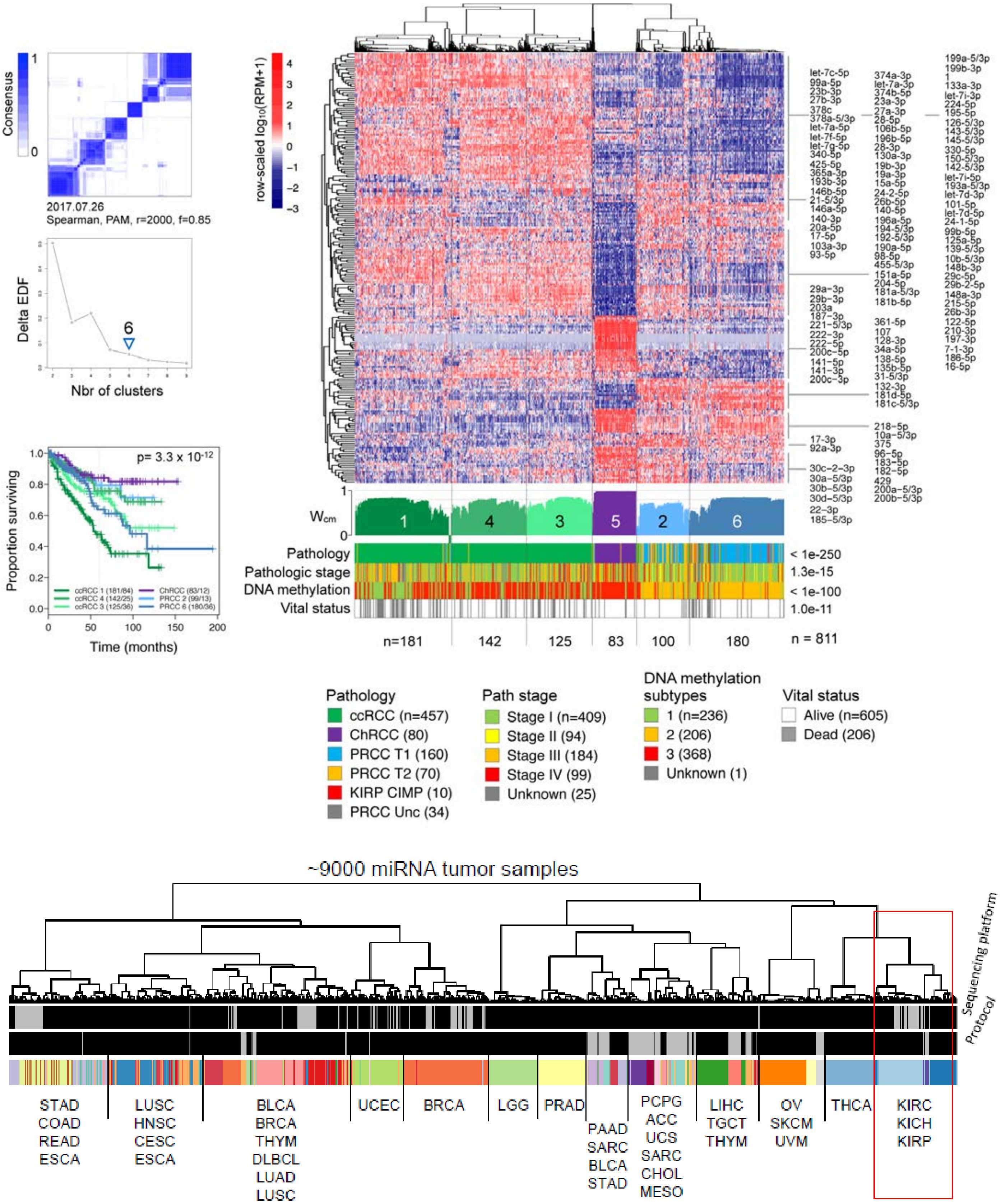

Figure S1

Figure S1C

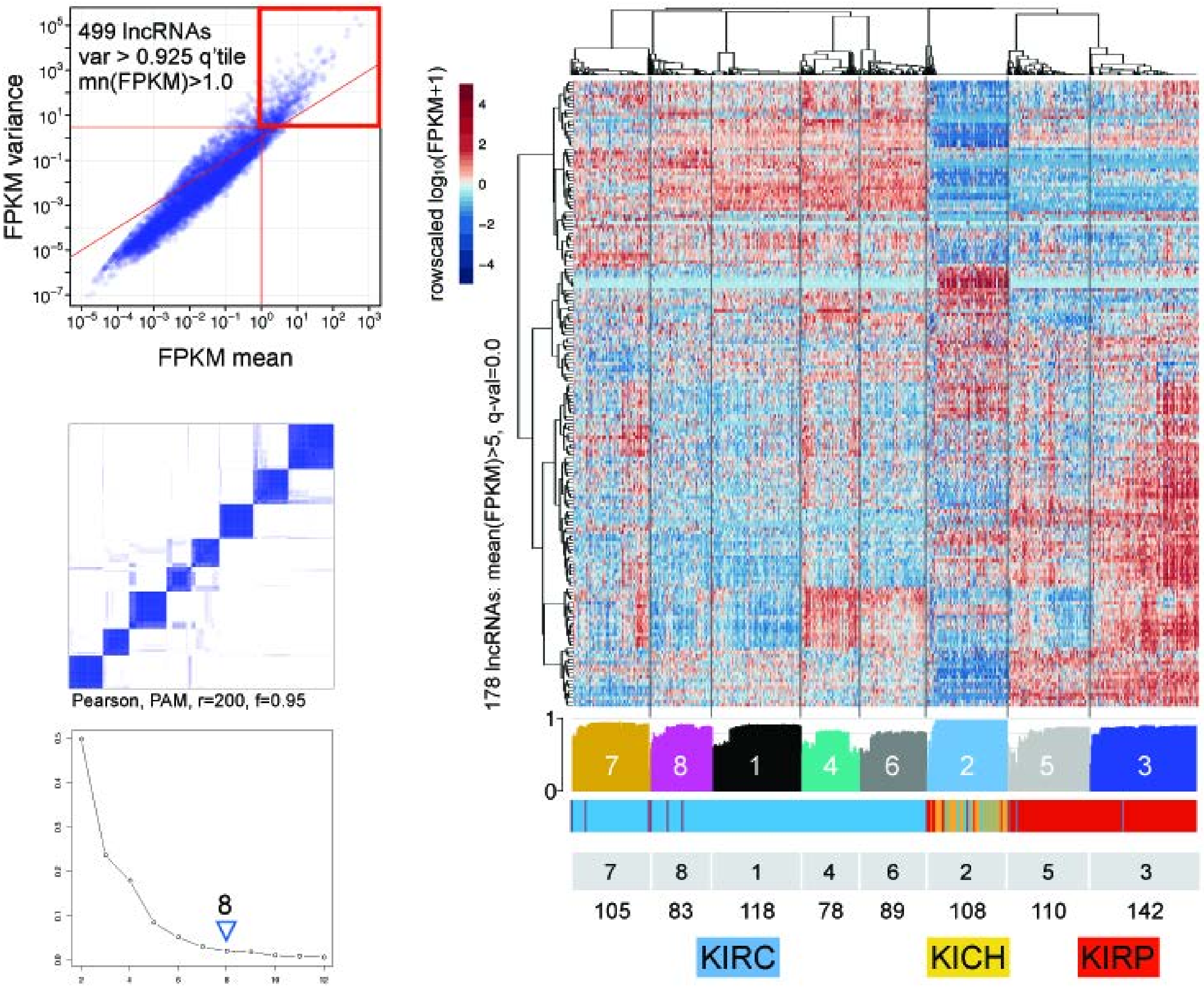

Figure S1

Figure S1 related to Figure 1: Cluster analysis of tumor mRNA, miRNA, and lncRNA

A. An mRNA consensus membership heatmap for 11,000 most variable genes identified kmeans consensus matrix for a 6-cluster solution. Clusters 1 and 2 were enriched for ccRCC, cluster 3 and 6 were enriched for PRCC, and cluster 5 was enriched for ChRCC. Cluster 4 contained a mixture of histologies including ccRCC, Type 2 PRCC, ChRCC, and the majority of CIMP-RCC. Overall survival was compared between clusters using a Log-rank test.

B. Unsupervised consensus clustering of miRNA expression profiles for 811 Pan-Kidney primary tumor samples. The input was RPMs taken from from a batch-corrected Pan-Cancer dataset of 734 mature strands, for 367 miRNA-seq mature strands that were the union of the most-variable 25% in freeze members from each original primary tumor cohort. A consensus membership heatmap for an 6-cluster solution, with a 'delta' plot, was used to produce a normalized abundance heatmap for the 6-cluster solution. Below the heatmap is a silhouette width profile (Wcm) calculated from the consensus membership, then covariate tracks, then a table of cluster membership. miRNAs listed to the right are a subset of the 213 in the heatmap. Association p values to the right of the covariate tracks are Chi-square or Fisher exact, uncorrected for multiple testing. A Kaplan-Meier plot for overall survival is shown. Comparison of miRNA profiles across the ~9,000 PanCanAtlas samples demonstrated a distinct separation of RCC samples from other cancers.

C. An unsupervised consensus clustering of lncRNA expression profiles for 833 pan-kidney tumor samples was produced using lncRNA profiles calculated from RNAseq data using Ensembl v82 (Sept 2015) gene annotations. A selection of 499 highly variant lncRNAs were used to create a consensus membership heatmap for an 8-cluster solution, with a 'delta' plot. A normalized abundance (FPKM) heatmap for the 8-cluster solution was produced for 178 highly variant lncRNAs with mean FPKM > 5. Below the heatmap is a silhouette width profile calculated from the consensus membership, and a covariate track showing tumor types, with KIRC=blue, KICH=yellow and KIRP=red.

Figure S1

Figure S2A

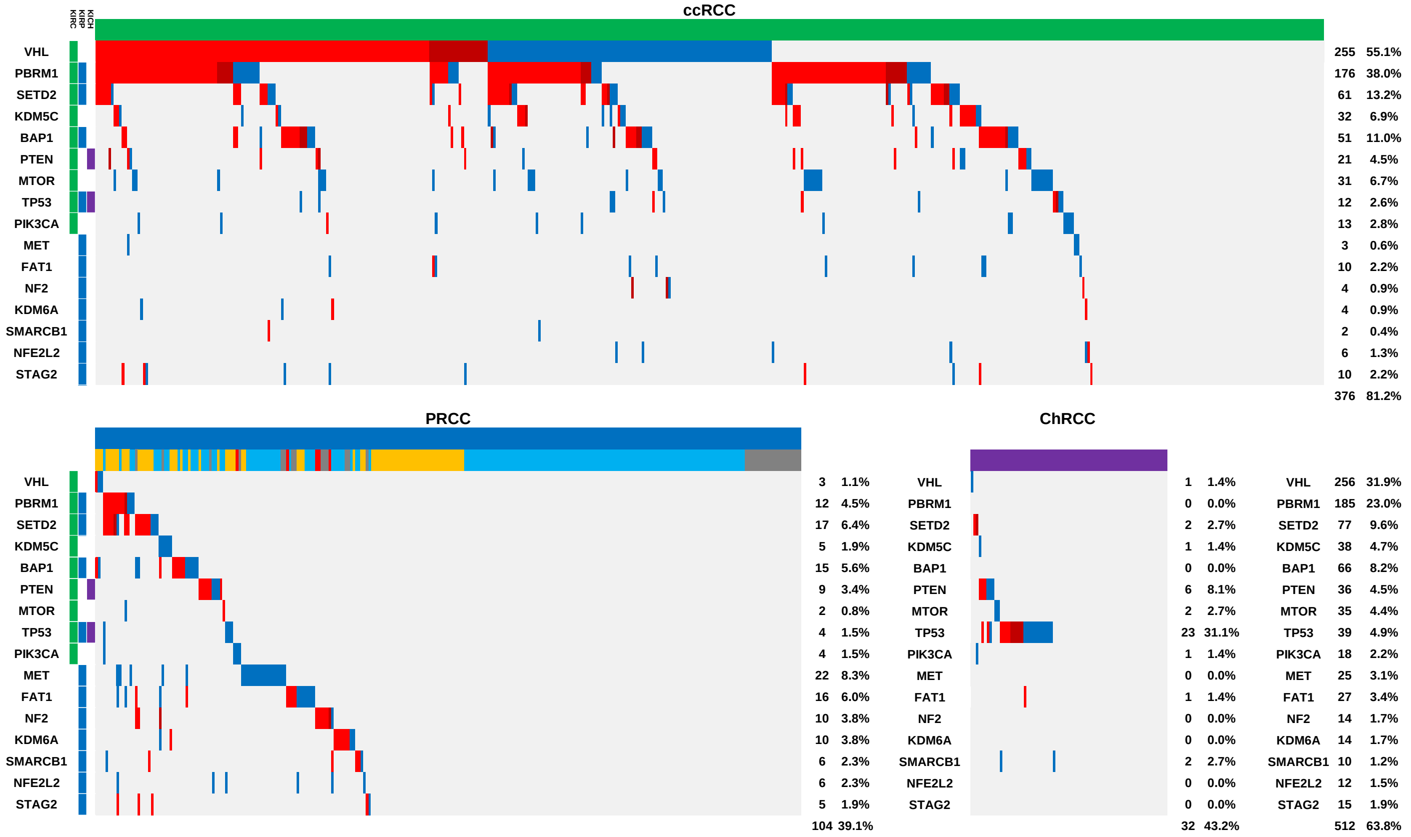

Figure S2B

Mutant vs Wild-Type

	All RCC	CCRCC	PRCC	ChRCC
VHL	0.0057	0.8414	0.5084	1.0000
PBRM1	0.0478	0.5268	0.0008	-
SETD2	0.0089	0.3061	0.0189	0.2705
KDM5C	0.0909	0.1147	0.3470	0.6730
BAP1	0.0002	0.0035	0.6081	-
PTEN	0.2526	0.4101	0.7078	0.0138
MTOR	0.1029	0.2771	0.7171	0.5914
TP53	0.1136	0.0002	0.0049	0.0876
PIK3CA	0.2643	0.6909	0.0117	0.0276
MET	0.5559	0.9192	0.9371	-
FAT1	0.5375	0.6963	0.8187	0.6950
NF2	0.4548	0.8390	0.2256	-
KDM6A	0.4725	0.1545	0.8366	-
SMARCB1	0.9240	0.1484	0.2748	0.3036
NFE2L2	0.9699	0.3141	0.2619	-
STAG2	0.8157	0.6030	0.9078	-

Figure S2

Figure S2C

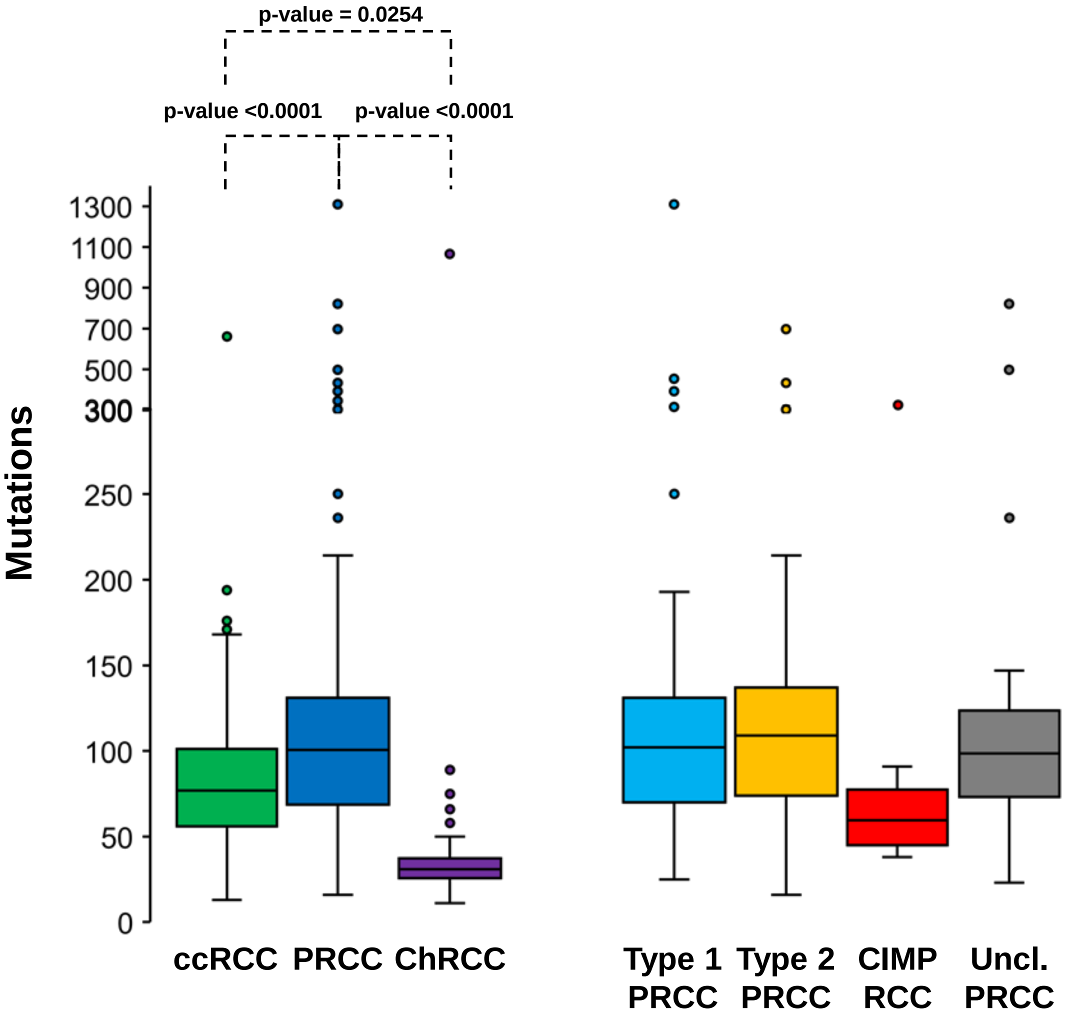

Figure S2D

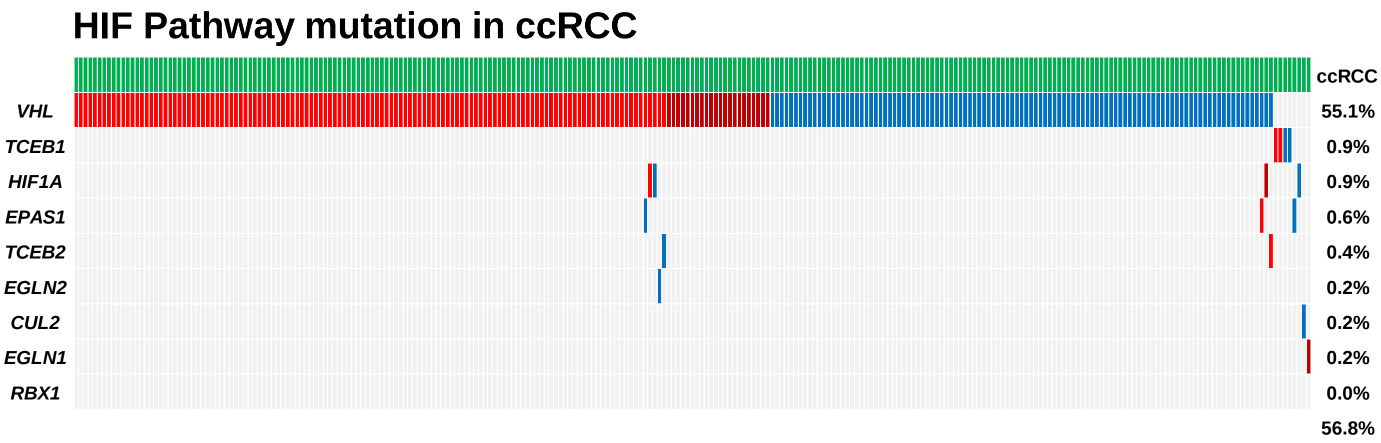

Figure S2

Figure S2D (cont.)

HIPPO Signaling Pathway

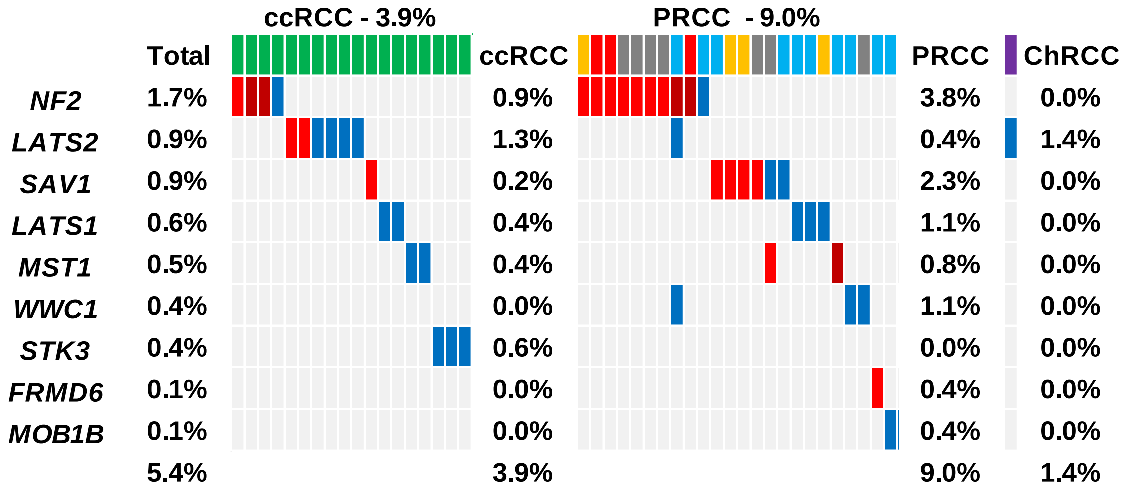

NRF2/ARE (antioxidant responsive element) Pathway

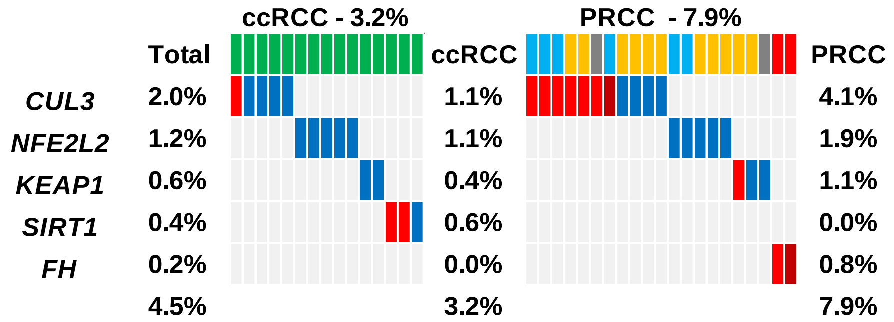

PI3K/AKT Pathway

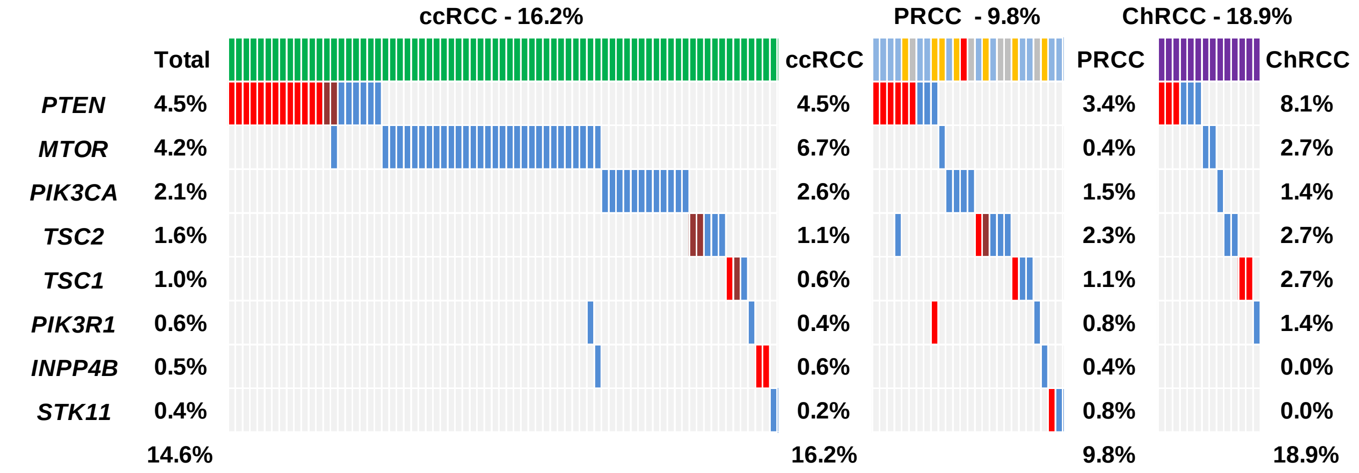

Figure S2D (cont.)

Chromatin Remodeling Pathways

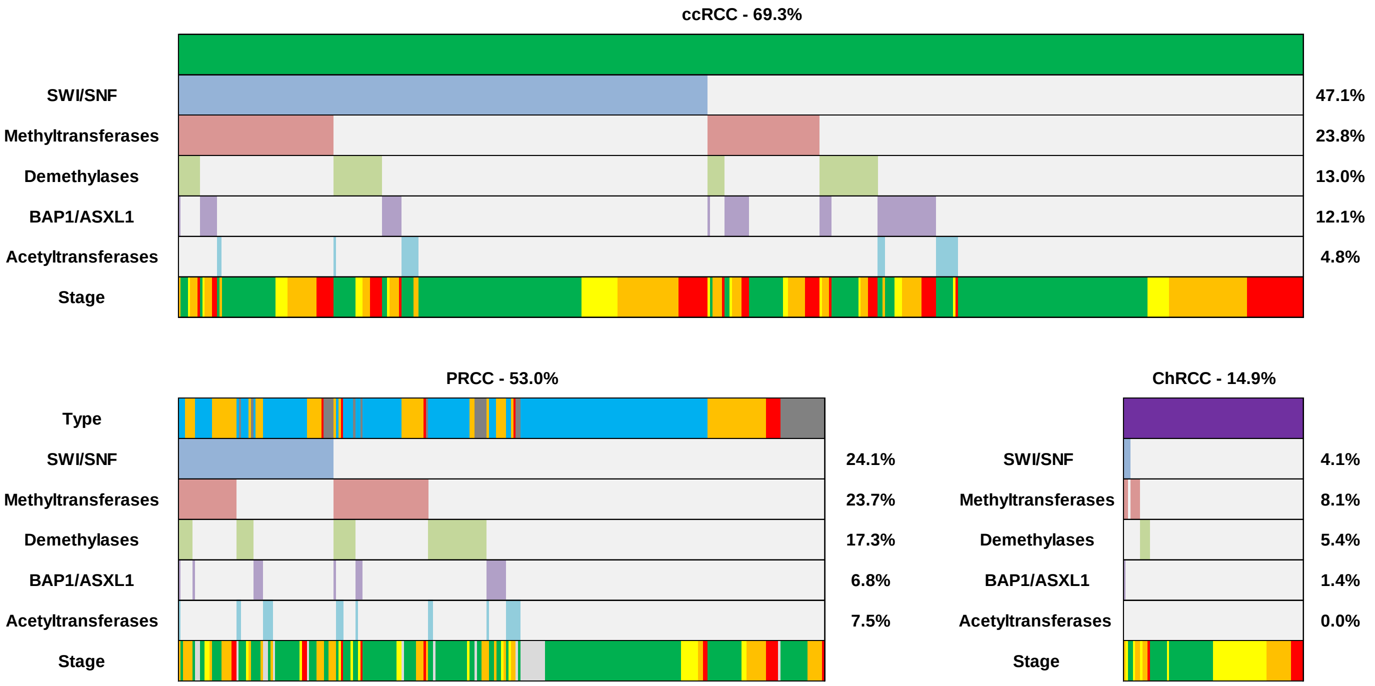

Figure S2E

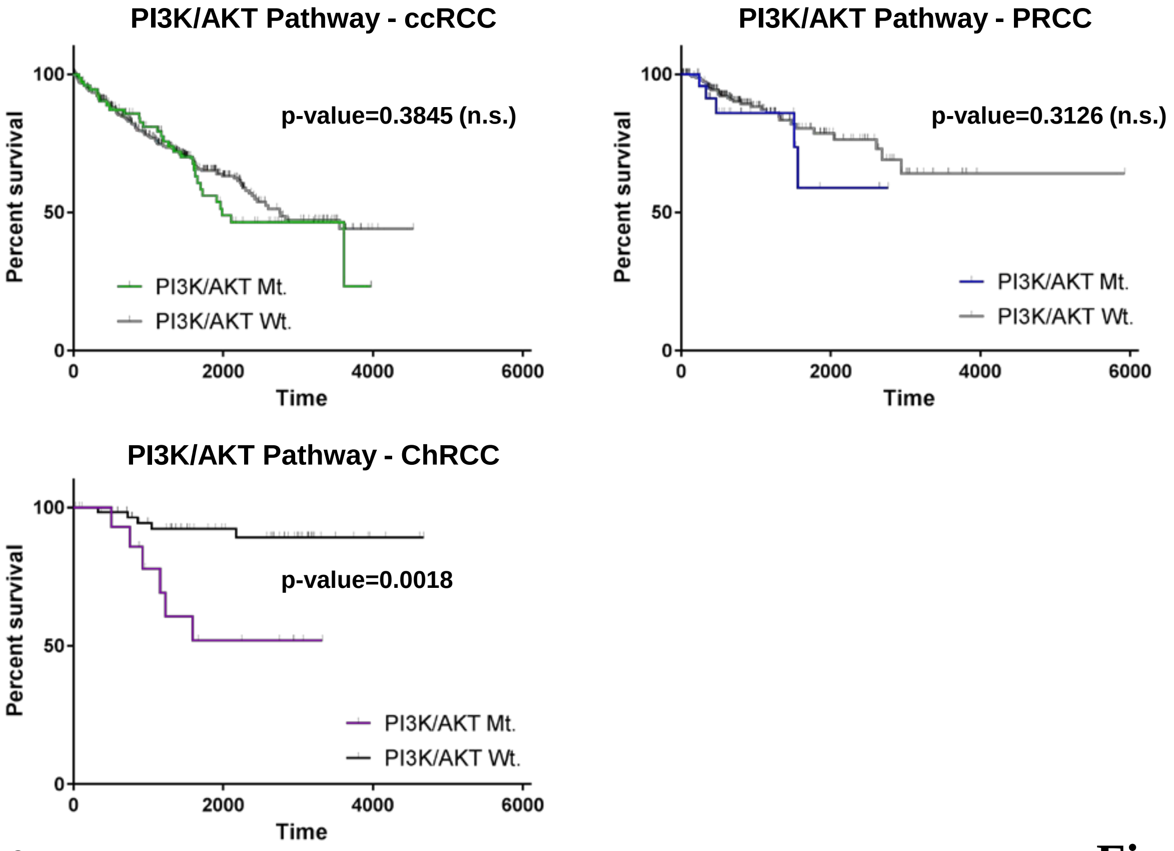

Figure S2F

Category		Mutations	
1	No pathogenic mutation	- No somatic mutations	
2		- low and medium heteroplasmy D-loop and "non-coding-region" mutations<br>- low heteroplasmy tRNA and rRNA mutations<br>- low heteroplasmy missense mutations	
3		- high heteroplasmy D-loop mutations<br>- medium and high heteroplasmy tRNA and rRNA mutations<br>- medium heteroplasmy missense and nonsense mutations<br>- frameshift mutations below 50% mutation load	3 or more mutations of category 2
4	Mutated	- high heteroplasmy missense mutations	3 or more mutations of category 3
5		- Frameshift mutations above 50% mutation load<br>- High heteroplasmy nonsense mutations	3 or more mutations of category 4

Low heteroplasmy
<25% mutational load

Medium heteroplasmy
25-75% mutational load

High heteroplasmy
>75% mutational load

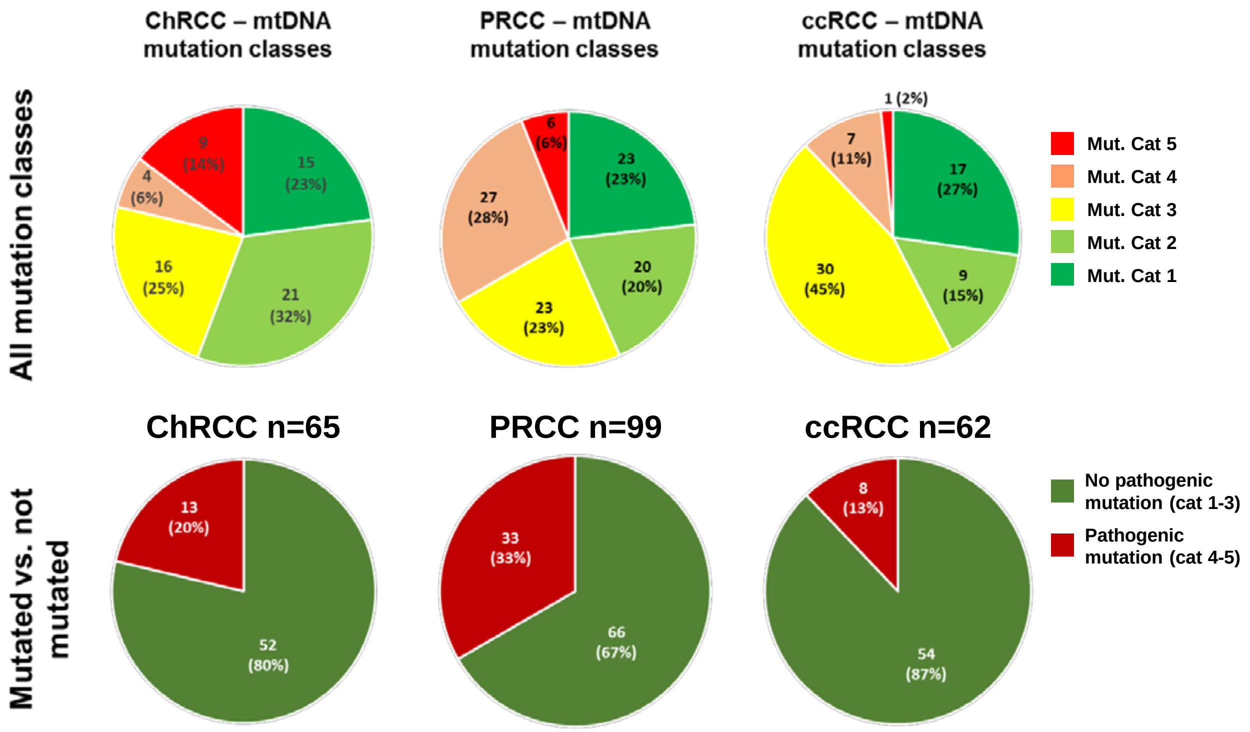

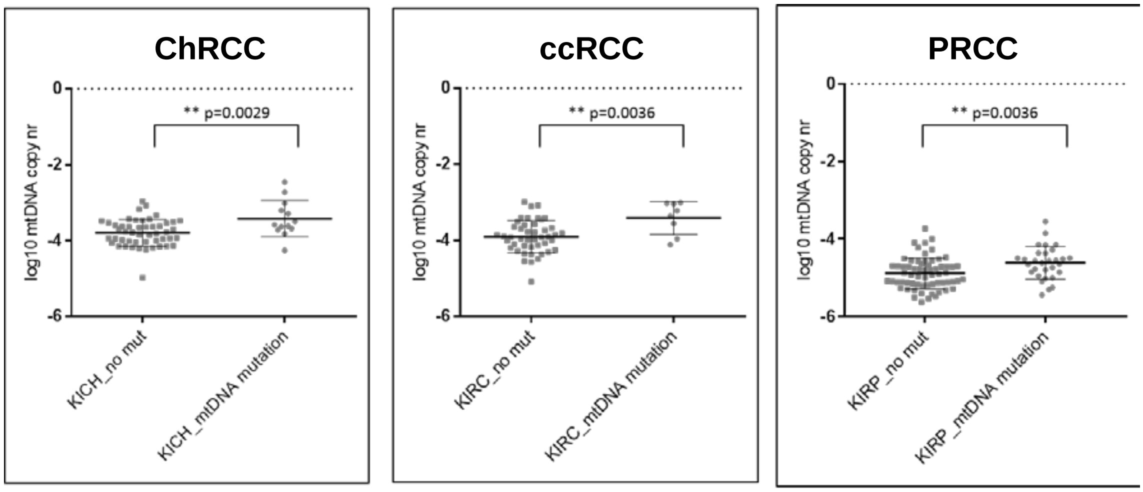

mtDNA copy number is significantly increased in samples with pathogenic mtDNA mutations (unpaired t-test)

Figure S2

Figure S2 related to Figure 2: Somatic and Mitochondrial Mutation Analysis

A. An oncoprint for the 16 significantly mutated genes identified across the three individual analyses of the TCGA KIRC (indicated in green), KIRP (indicated in blue), and KICH (indicated in purple) projects. Mutations were colored blue for missense mutations and in-frame insertion/deletions, crimson for splice mutations, and red for nonsense and frameshift insertion/deletions. Number and percentage of mutation are shown for each RCC subtype and in total. The PRCC samples are sub-classified into Type 1 PRCC (light blue), Type 2 PRCC (orange), CIMP-RCC (red), and unclassified PRCC (gray).
B. Overall survival comparing samples with and without SMG mutation both within the major RCC subtypes and across the cohort were calculated using a Log-rank test and tabulated.
C. A box and whisker plot of the mutation number per tumor for the three major RCC subtypes, ccRCC (green), PRCC (blue), and ChRCC (purple) and the PRCC subtypes, Type 1 PRCC (light blue), Type 2 PRCC (orange), CIMP-RCC (red), and unclassified PRCC (gray). Mutation number was compared between RCC subtypes using a T-test.
D. Oncoprints for the HIF pathway, HIPPO signaling pathway, NRF2/ARE pathway, PI3K/AKT pathway, and the chromatin remodeling pathways. Mutations were colored blue for missense mutations and in-frame insertion/deletions, crimson for splice mutations, and red for nonsense and frameshift insertion/deletions. The three major RCC subtypes were color-coded green for ccRCC, blue for PRCC, and purple for ChRCC and the PRCC samples were sub-classified into Type 1 PRCC (light blue), Type 2 PRCC (orange), CIMP-RCC (red), and unclassified PRCC (gray).
E. Overall survival comparing samples with and without PI3K/AKT pathway mutation within the major RCC subtypes was calculated using a Log-rank test.
F. Somatic mitochondrial mutations were categorized by amino-acid change, frequency, and degree of heteroplasmy. Piecharts were produced to demonstrate the breakdown of mtDNA mutation classes within the three major RCC subtypes and the percentage of significant mutations. Comparisons of mitochondrial genome copy number between mutated and non-mutated samples were performed using unpaired T-tests and demonstrated significantly higher amounts of mtDNA within the mutated samples.

Figure S2

Figure S3A

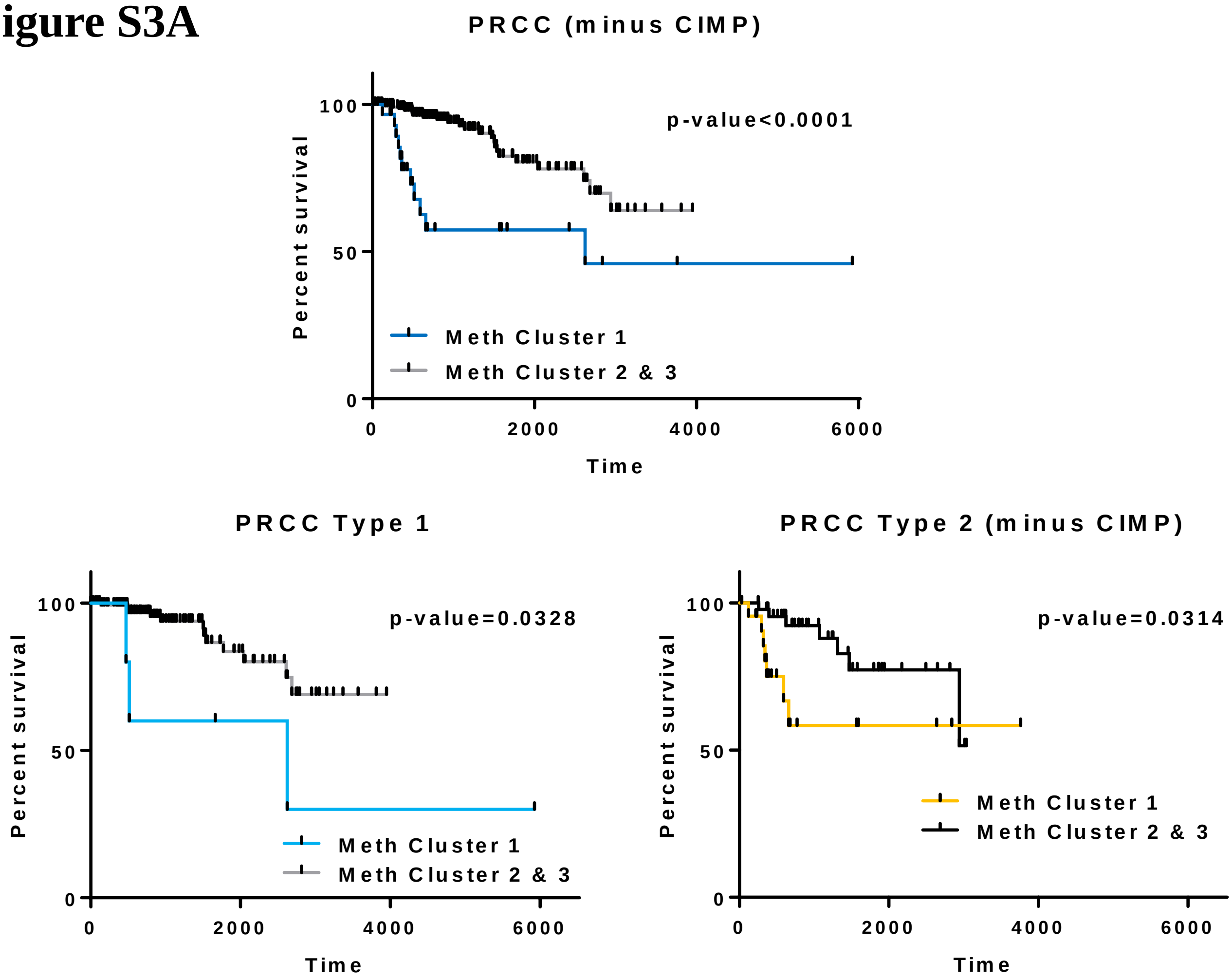

Figure S3B

Fisher's exact test in ccRCC for stage vs. methylation

	Stage I-II	Stage III-IV	Total
Cluster 1	70	112	182
Cluster 2 & 3	204	84	288
Total	274	196	470

The two-tailed P value is less than 0.0001

Fisher's exact test in PRCC for stage vs. methylation (without CIMP samples)

	Stage I-II	Stage III-IV	Total
Cluster 1	7 (6)	33 (24)	40 (30)
Cluster 2 & 3	178	31	209
Total	185 (184)	64 (55)	249 (239)

The two-tailed P value is less than 0.0001 both with and without CIMP

Fisher's exact test in ChRCC for stage vs. methylation

	Stage I-II	Stage III-IV	Total
Cluster 1	3	13	16
Cluster 2 & 3	52	12	64
Total	55	25	80

The two-tailed P value is less than 0.0001

Fisher's exact test in ccRCC for *SETD2* mutation vs. methylation

	SETD2 Mt.	*SETD2* Wt.	Total
Cluster 1	43	134	177
Cluster 2 & 3	17	257	274
Total	60	391	451

The two-tailed P value is less than 0.0001

Fisher's exact test in Type 2 PRCC for *PBRM1* mutation vs. methylation

	PBRM1 Mt.	*PBRM1* Wt.	Total
Cluster 1	7	23	30
Cluster 2 & 3	1	45	46
Total	8	68	76

The two-tailed P value equals 0.0053

Fisher's exact test in Type 2 PRCC for *SETD2* mutation vs. methylation

	SETD2 Mt.	*SETD2* Wt.	Total
Cluster 1	9	21	30
Cluster 2 & 3	4	42	46
Total	13	63	76

The two-tailed P value equals 0.0270

Fisher's exact test in Type 2 PRCC for *SETD2/PBRM1* mutation vs. methylation

	SETD2/PBRM1 Mt.	*SETD2/PBRM1* Wt.	Total
Cluster 1	10	20	30
Cluster 2 & 3	4	42	46
Total	14	62	76

The two-tailed P value equals 0.0133

Fisher's exact test in ChRCC for *TP53* mutation vs. methylation

	TP53 Mt.	*TP53* Wt.	Total
Cluster 1	9	7	16
Cluster 2 & 3	14	50	64
Total	23	57	80

The two-tailed P value equals 0.0119

Figure S3

Figure S3C

DKK1 – cg07684796 *SFRP1* – cg15839448

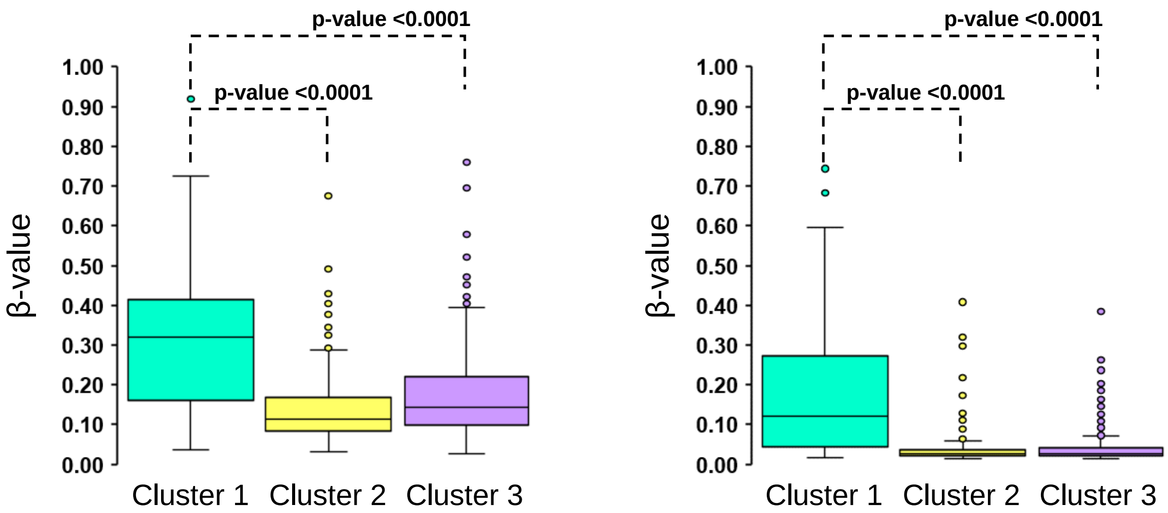

Figure S3D

PRCC *DKK1/SFRP1* Methylation (minus CIMP)

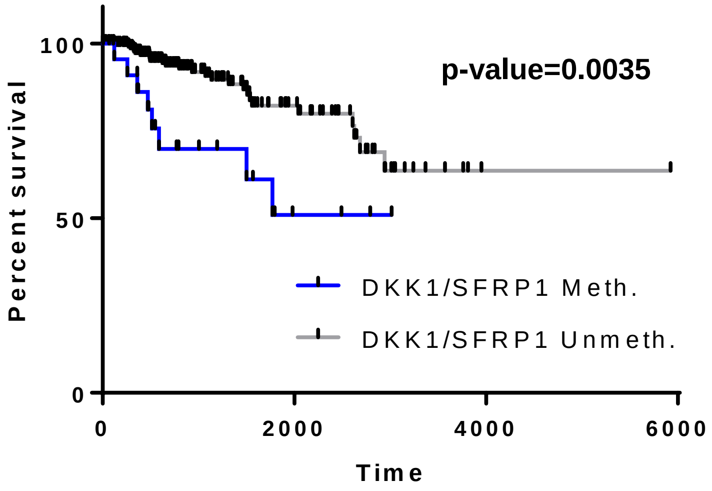

Figure S3 related to Figure 3: Methylation Cluster Analysis

A. Overall survival comparing samples within methylation cluster 1 to samples in other methylation clusters for PRCC and the Type 1 and Type 2 PRCC subtypes using a Log-rank test.
B. Association of stage and somatic mutation of *SETD2*, *PBRM1*, and *TP53* with methylation cluster patterns using Fisher's exact testing.
C. Box and whisker plots of the β–values for two selected Illumina probes within the CpG islands of the *DKK1* and *SFRP1* genes across the three methylation clusters, cluster 1 (light blue), cluster 2 (yellow), and cluster 3 (light purple). Comparison of β–values across clusters was performed using a T-test.
D. Overall survival comparing samples with and without *DKK1*/*SFRP1* methylation within the PRCC tumors minus the CIMP-RCC samples calculated using a Log-rank test.

Figure S3

Figure S4A

Electron Transport Chain (ETC) Complex

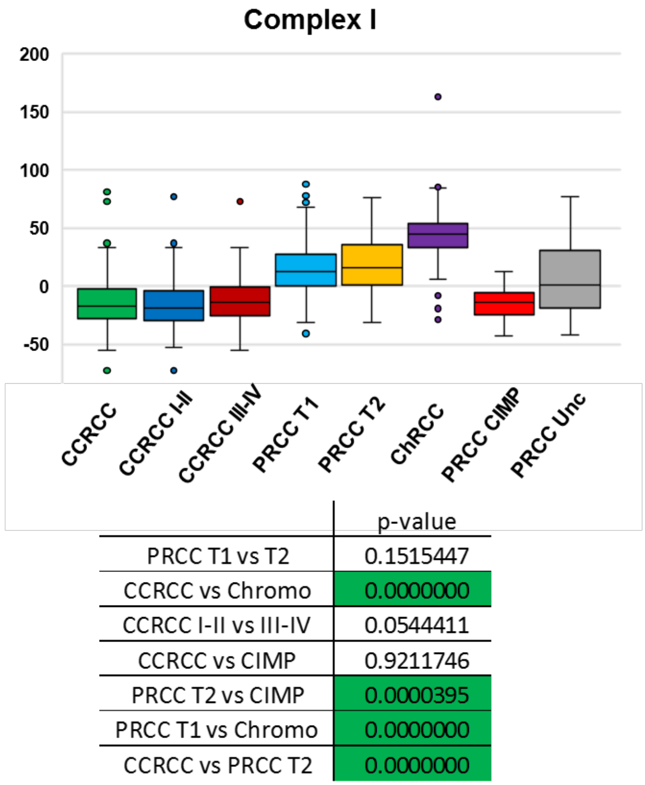

	p-value
PRCC T1 vs T2	0.1515447
CCRCC vs Chromo	0.0000000
CCRCC I-II vs III-IV	0.0544411
CCRCC vs CIMP	0.9211746
PRCC T2 vs CIMP	0.0000395
PRCC T1 vs Chromo	0.0000000
CCRCC vs PRCC T2	0.0000000

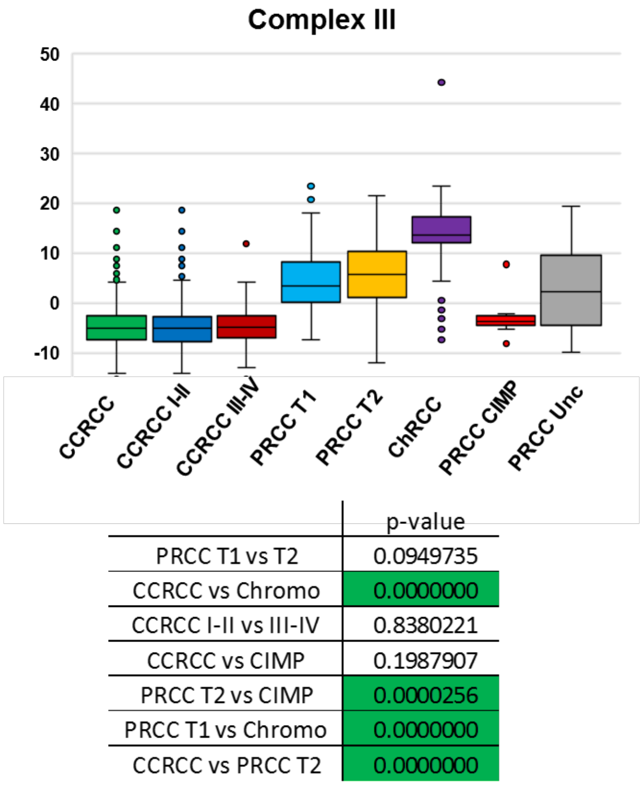

	p-value
PRCC T1 vs T2	0.0949735
CCRCC vs Chromo	0.0000000
CCRCC I-II vs III-IV	0.8380221
CCRCC vs CIMP	0.1987907
PRCC T2 vs CIMP	0.0000256
PRCC T1 vs Chromo	0.0000000
CCRCC vs PRCC T2	0.0000000

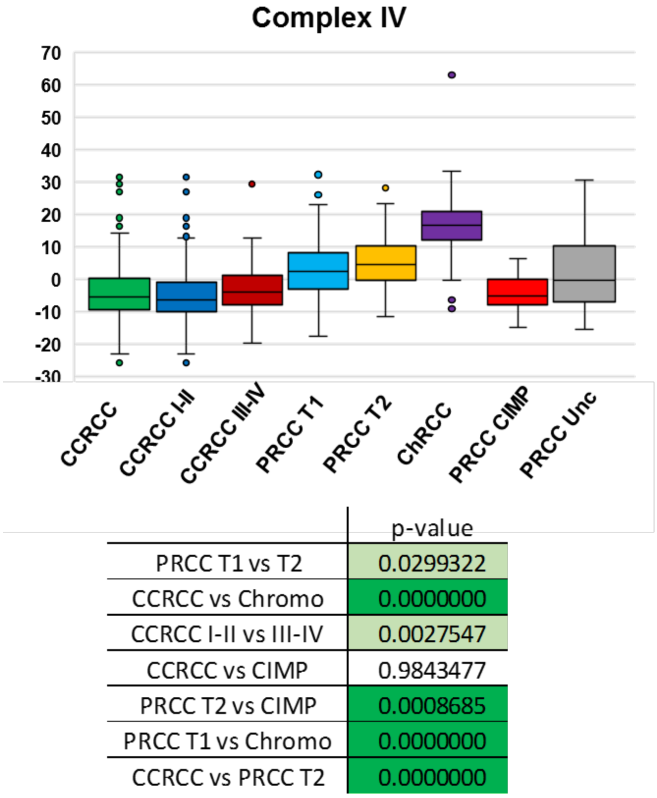

	p-value
PRCC T1 vs T2	0.0299322
CCRCC vs Chromo	0.0000000
CCRCC I-II vs III-IV	0.0027547
CCRCC vs CIMP	0.9843477
PRCC T2 vs CIMP	0.0008685
PRCC T1 vs Chromo	0.0000000
CCRCC vs PRCC T2	0.0000000

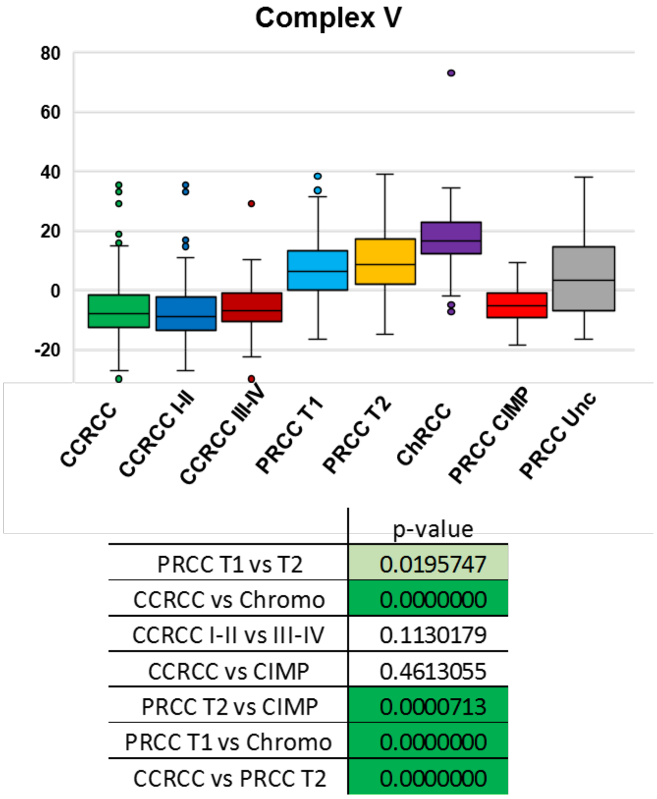

	p-value
PRCC T1 vs T2	0.0195747
CCRCC vs Chromo	0.0000000
CCRCC I-II vs III-IV	0.1130179
CCRCC vs CIMP	0.4613055
PRCC T2 vs CIMP	0.0000713
PRCC T1 vs Chromo	0.0000000
CCRCC vs PRCC T2	0.0000000

Figure S4A (cont.)

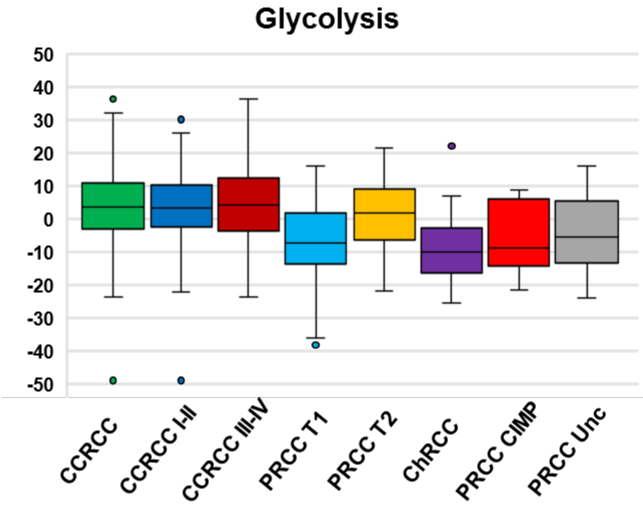

	p-value
PRCC T1 vs T2	0.0000001
CCRCC vs Chromo	0.0000000
CCRCC I-II vs III-IV	0.5380227
CCRCC vs CIMP	0.0275195
PRCC T2 vs CIMP	0.1048725
PRCC T1 vs Chromo	0.0594869
CCRCC vs PRCC T2	0.0317904

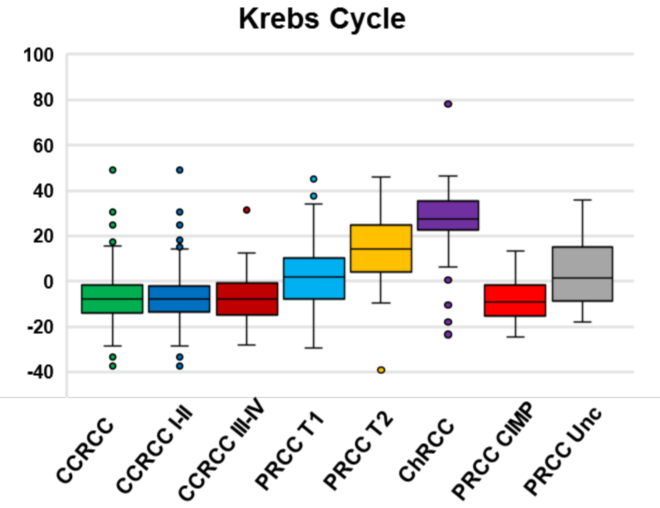

	p-value
PRCC T1 vs T2	0.0000001
CCRCC vs Chromo	0.0000000
CCRCC I-II vs III-IV	0.2791155
CCRCC vs CIMP	0.7832211
PRCC T2 vs CIMP	0.0000483
PRCC T1 vs Chromo	0.0000000
CCRCC vs PRCC T2	0.0000000

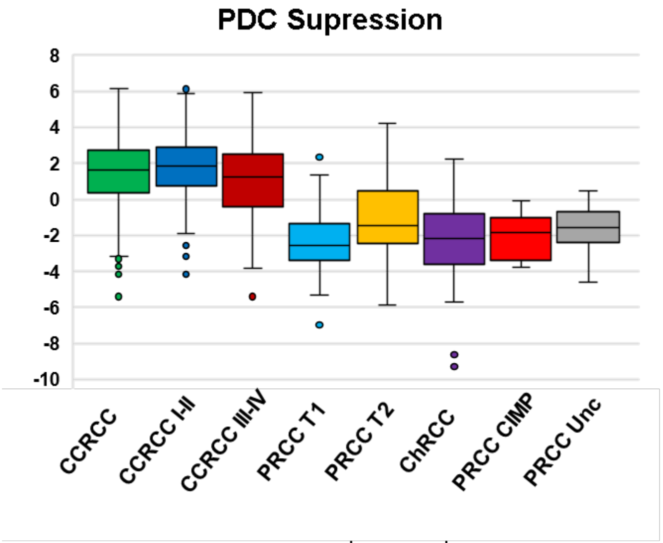

	p-value
PRCC T1 vs T2	0.0000130
CCRCC vs Chromo	0.0000000
CCRCC I-II vs III-IV	0.0000274
CCRCC vs CIMP	0.0000116
PRCC T2 vs CIMP	0.0845623
PRCC T1 vs Chromo	0.6202750
CCRCC vs PRCC T2	0.0000000

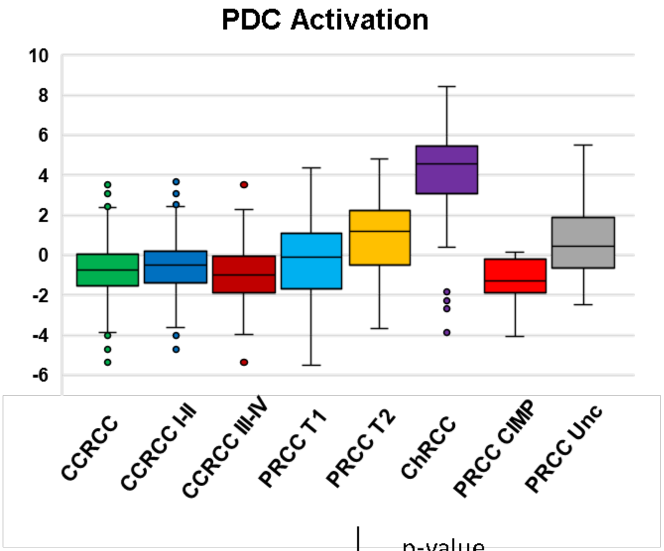

	p-value
PRCC T1 vs T2	0.0000599
CCRCC vs Chromo	0.0000000
CCRCC I-II vs III-IV	0.0004921
CCRCC vs CIMP	0.2563383
PRCC T2 vs CIMP	0.0002397
PRCC T1 vs Chromo	0.0000000
CCRCC vs PRCC T2	0.0000000

Figure S4A (cont.)

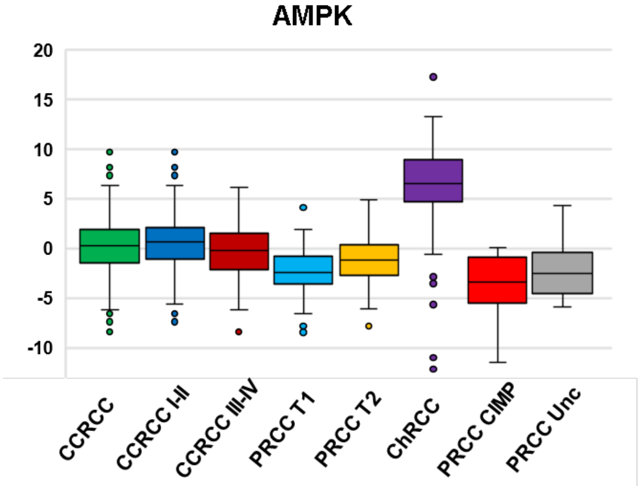

	p-value
PRCC T1 vs T2	0.0057894
CCRCC vs Chromo	0.0000000
CCRCC I-II vs III-IV	0.0005488
CCRCC vs CIMP	0.0051487
PRCC T2 vs CIMP	0.0483568
PRCC T1 vs Chromo	0.0000000
CCRCC vs PRCC T2	0.0000120

Figure S4B

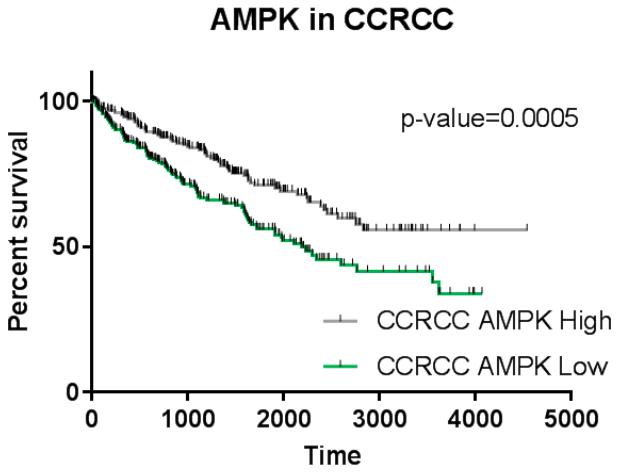

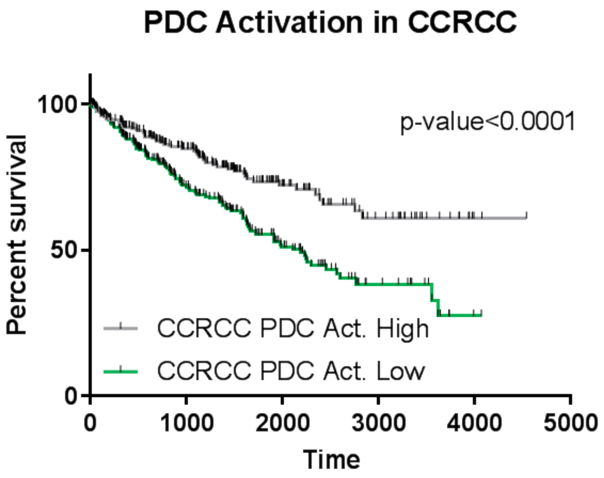

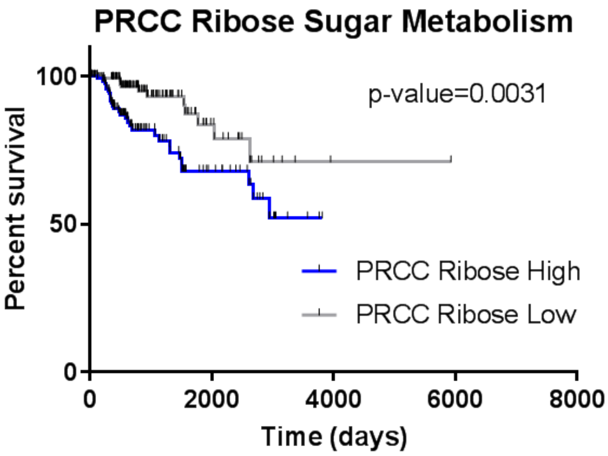

Figure S4 related to Figure 5: RCC Tumor Metabolic Analysis

A. Box and whisker plots for metabolic gene signatures for the three major RCC subtypes, ccRCC (green), PRCC (blue), and ChRCC (purple), high (crimson) and low (dark blue) stage ccRCCs, and the PRCC subtypes, Type 1 PRCC (light blue), Type 2 PRCC (orange), CIMP-RCC (red), and unclassified PRCC (gray). Comparison of RCC subtypes for each metabolic signature was performed using a T-test.
B. Overall survival comparing the higher 50% expressing samples with the lower 50% expression samples for the AMPK, PDC activation and Ribose Sugar Metabolism gene signatures in ccRCC and PRCC were calculated using a Log-rank test.

Figure S4

Figure S5A

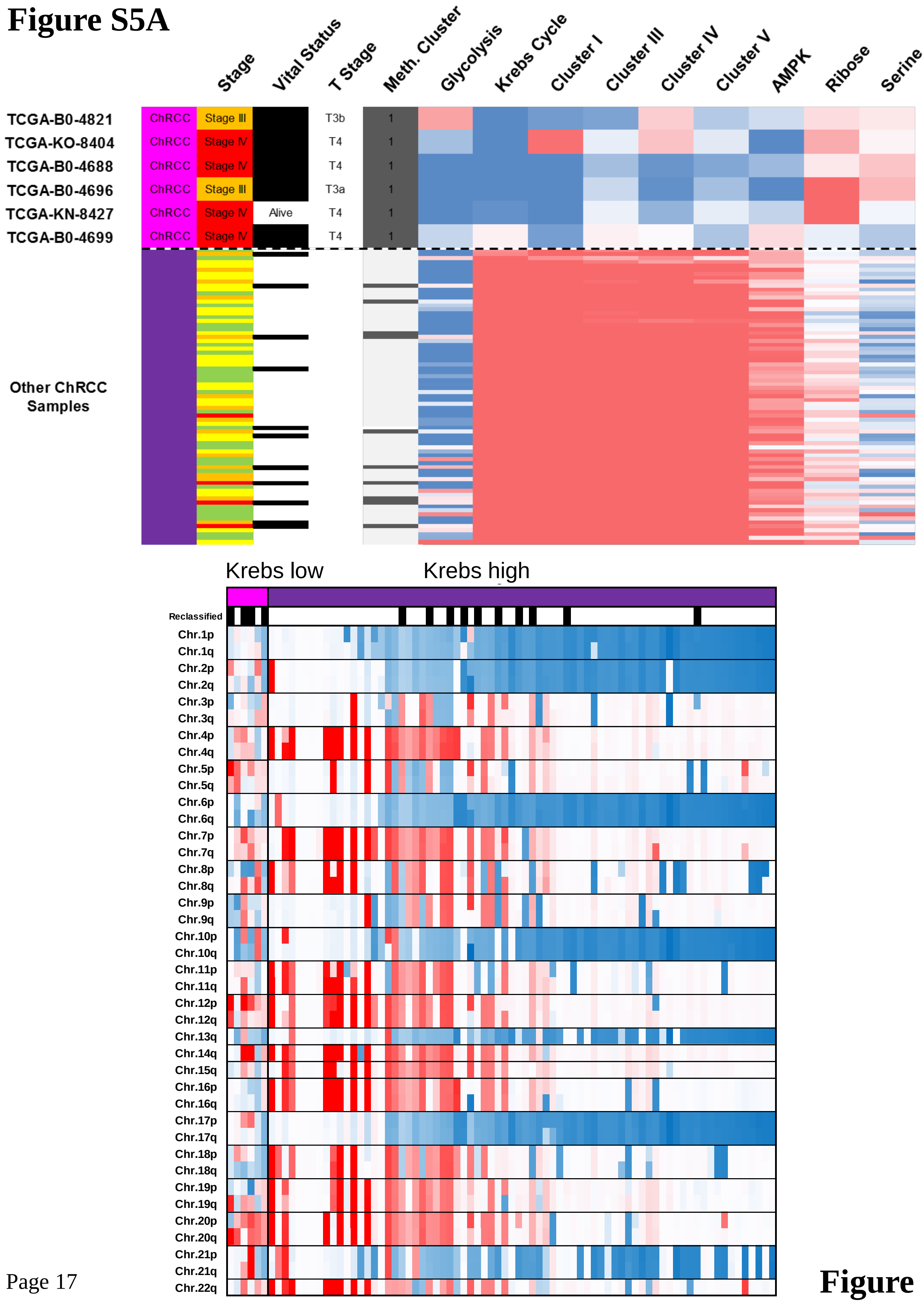

Figure S5

Figure S5B

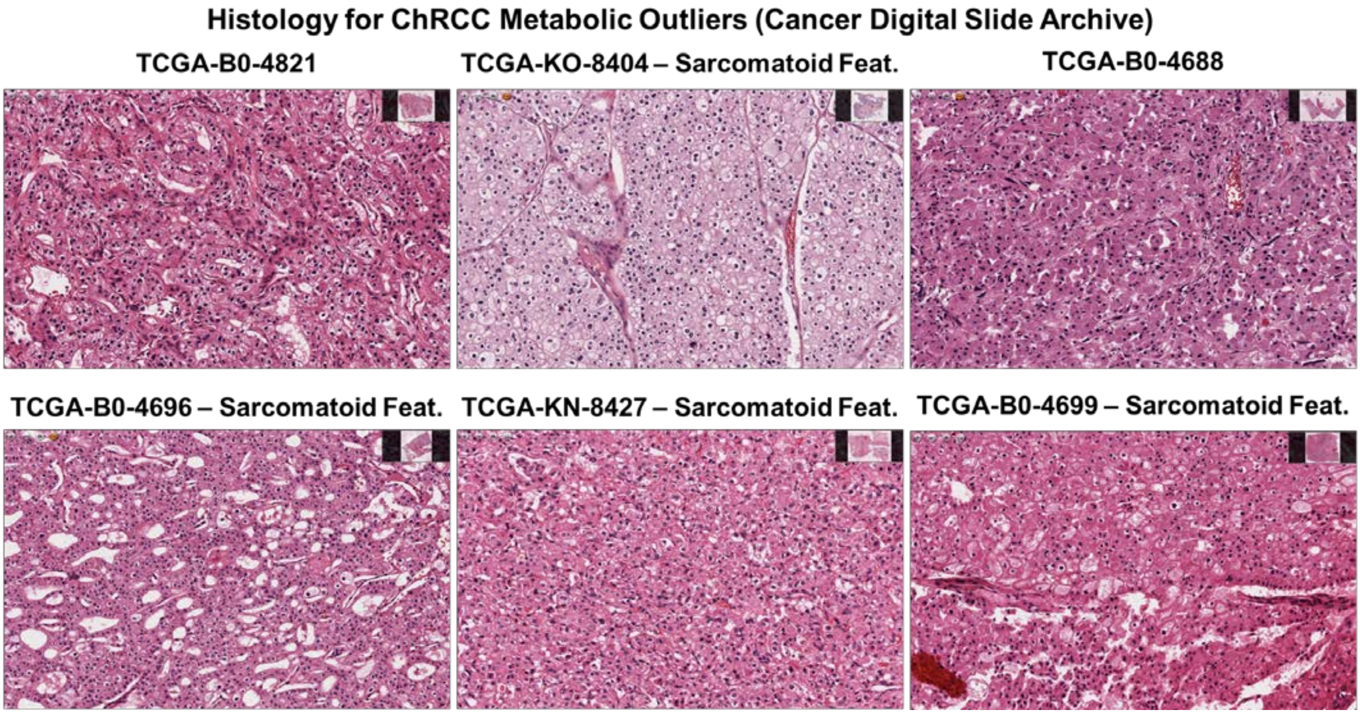

Figure S5 related to Figure 5: ChRCC Metabolic Analysis

A. Clinical, metabolic, and copy number data for the metabolically divergent ChRCC (Pink) in comparison to the remaining ChRCC samples (purple). The metabolic gene signatures demonstrated lower expression (blue) for the Krebs and ETC complex signatures. For copy number analysis, blue represents loss and red represents gain of chromosomal copy number
B. Pathology images downloaded from the Cancer Digital Slide Archive (http://cancer.digitalslidearchive.net/) for the the metabolically divergent ChRCC tumors.

Figure S6A

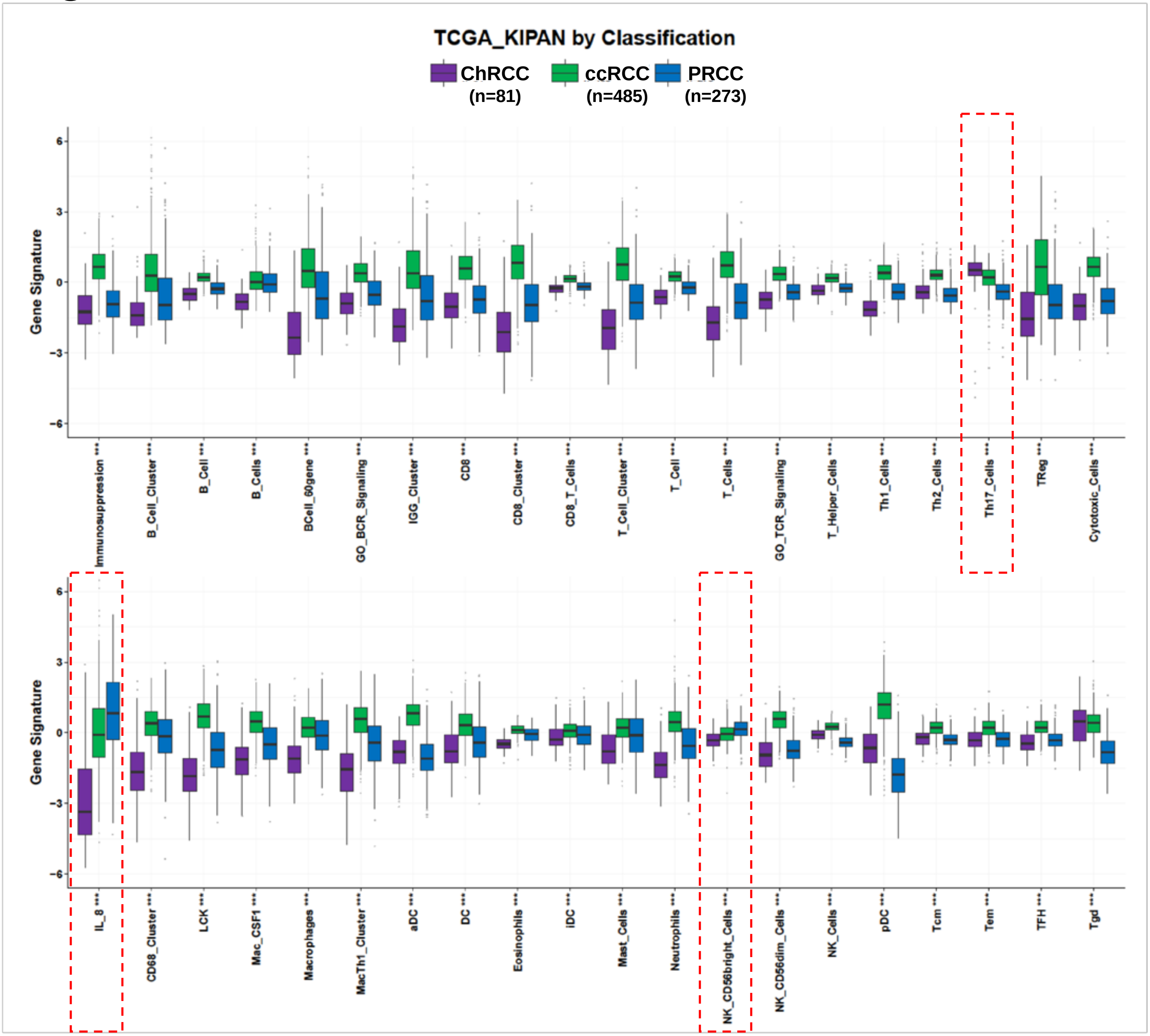

Figure S6

Figure S6B

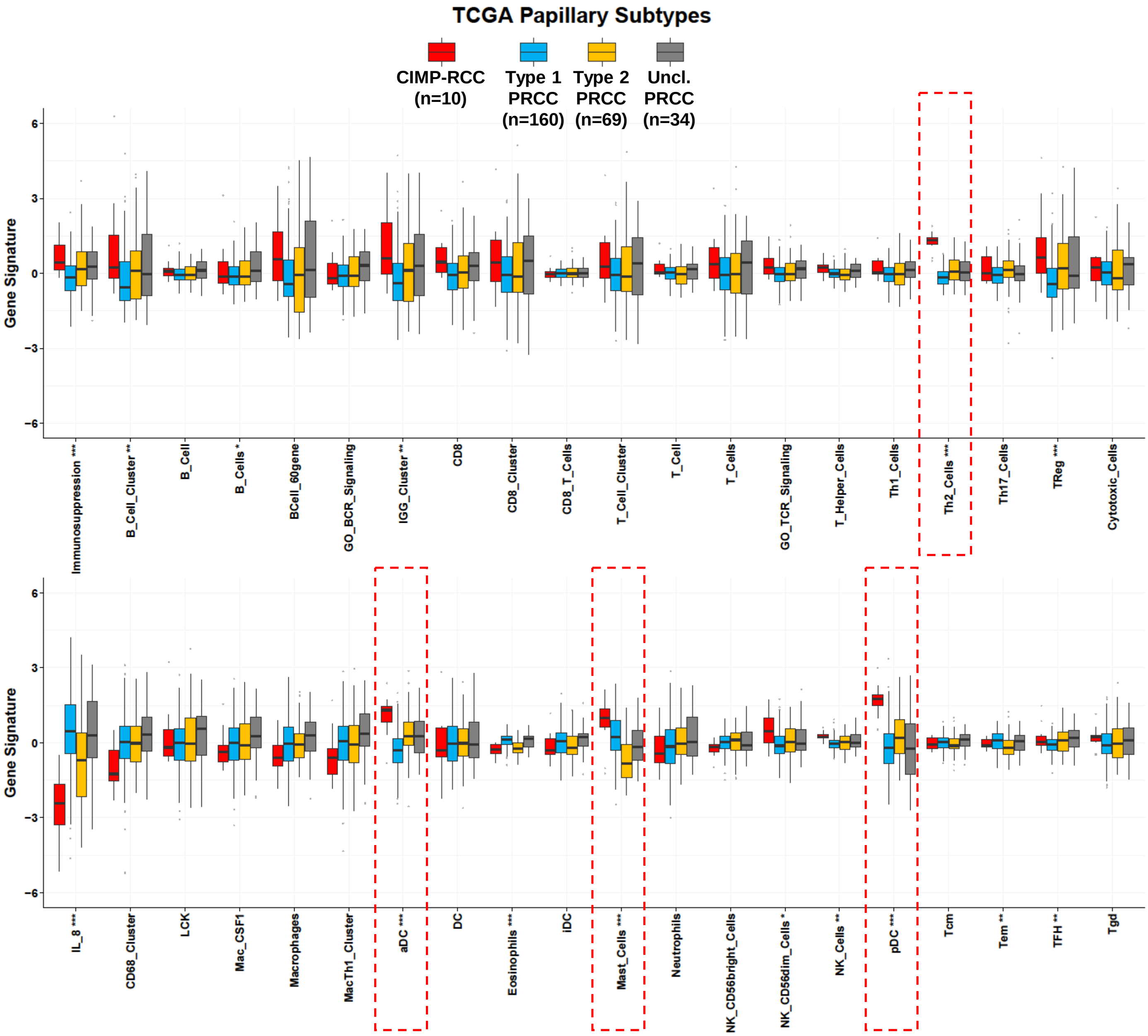

Figure S6

Figure S6C

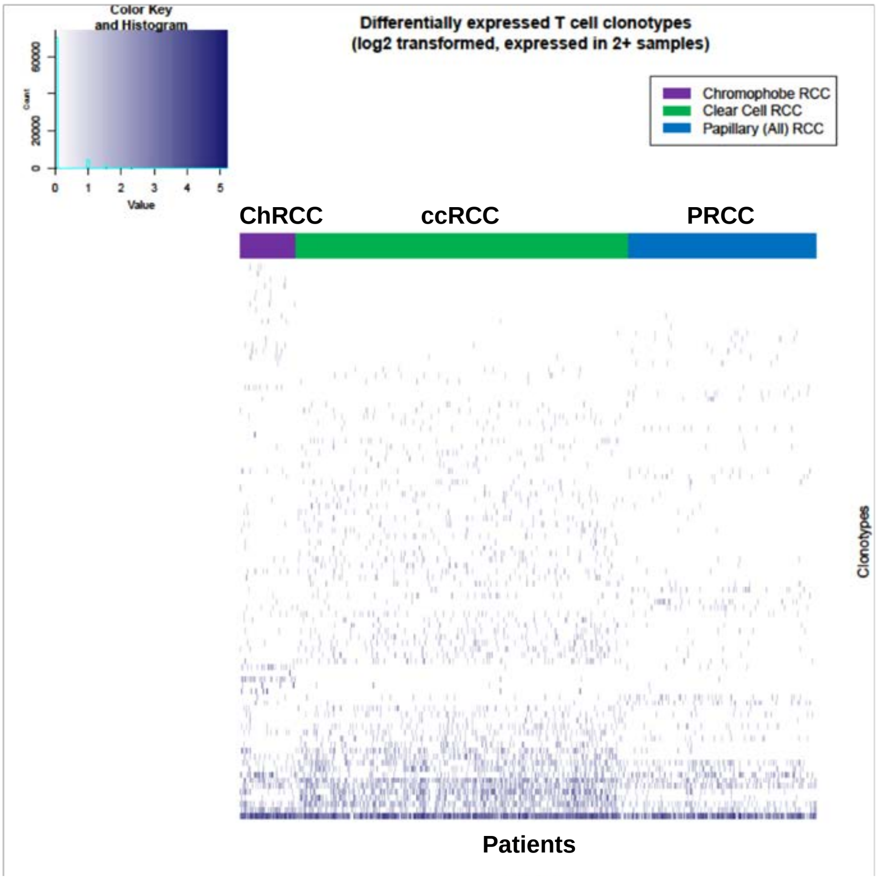

Figure S6D

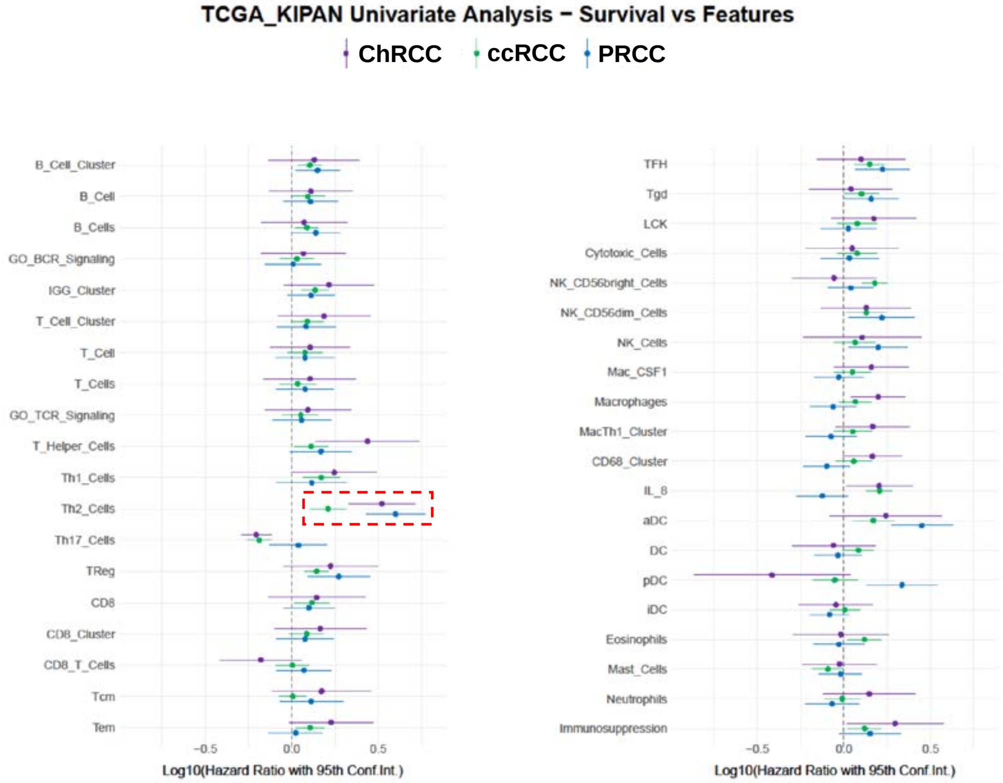

Figure S6

Figure S6E

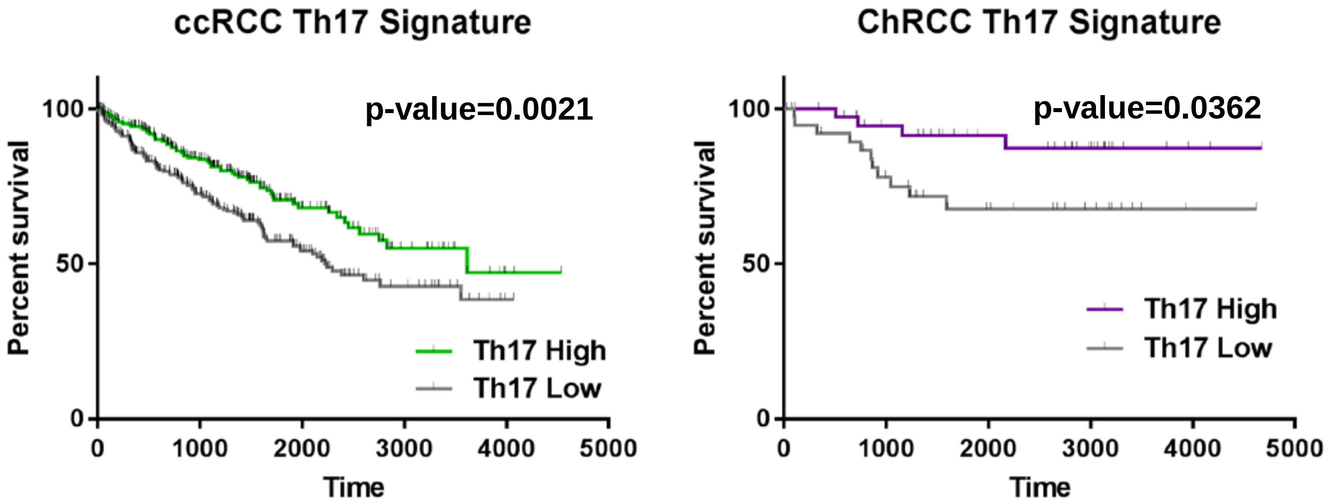

Figure S6 related to Figure 6: Immune Expression Profile Analysis

A. Expression of immune gene signatures were calculated from the RSEM (RNA-seq by expectancy maximization) upper quartile normalized gene expression data for ccRCC, PRCC, and ChRCC samples. Differential expression for each gene signature was analyzed between the major kidney cancer subtypes by 1-way analysis of variance and p-values were adjusted for multiple testing using the Benjamini-Hochberg procedure. Differentional expression of immune gene signatures demonstrated nearly ubiquitous upregulation in ccRCC compared to PRCC and ChRCC, except for the Th17, IL-8, and CD56bright NK cell gene signatures.
B. Differential expression for each gene signature was additionally analyzed between four defined PRCC subtypes consisting of 10 CIMP-RCC, 160 Type 1 PRCC, 69 Type 2 PRCC, and 34 Unclassified PRCC samples.
C. T cell receptor (TCR) profiling software MiXCR v1.7.1 was used to identify TCR clonotype expression in ccRCC, PRCC, and ChRCC samples. Differentially expressed TCR clonotypes (Fisher's exact testing) which were expressed in at least 2 samples are shown. Analysis demonstrated patterns of subtype specific TCR clonotype expression, suggesting possible variation in T cell response among ccRCC, PRCC, and ChRCC.
D. To determine the prognostic value of each immune gene signature, Cox proportional hazards (coxph) models were fit with signature expression value as the predictor and overall survival as the response variable. In accordance with previous findings, gene signatures which were significantly correlated with survival were largely associated with reduced survival, including those signatures which represented T cells, B cells, macrophages, dendritic cells, and NK cells.
E. Overall survival comparing the higher 50% expressing samples with the lower 50% expression samples for the Th17 immune gene signatures in ccRCC and ChRCC were calculated using a Log-rank test.

Figure S6
